# Selective citation in scientific literature on the human health effects of bisphenol A

**DOI:** 10.1186/s41073-019-0065-7

**Published:** 2019-03-29

**Authors:** M. J. E. Urlings, B. Duyx, G. M. H. Swaen, L. M. Bouter, M. P. Zeegers

**Affiliations:** 1 NUTRIM School of Nutrition and Translational Research in Metabolism, Maastricht University, Maastricht, The Netherlands; 2 Department of Epidemiology and Biostatistics, VU University Medical Center, Amsterdam, The Netherlands; 3 Department of Philosophy, Faculty of Humanities, Vrije Universiteit, Amsterdam, The Netherlands; 4 Care and Public Health Research Institute (CAPHRI), Maastricht University, Maastricht, The Netherlands

**Keywords:** Questionable research practice, Selective citation, Citation analysis, Methodology, Bisphenol A

## Abstract

**Introduction:**

Bisphenol A is highly debated and studied in relation to a variety of health outcomes. This large variation in the literature makes BPA a topic that is prone to selective use of literature, in order to underpin one’s own findings and opinion. Over time, selective use of literature, by means of citations, can lead to a skewed knowledge development and a biased scientific consensus. In this study, we assess which factors drive citation and whether this results in the overrepresentation of harmful health effects of BPA.

**Methods:**

A citation network analysis was performed to test various determinants of citation. A systematic search identified all relevant publications on the human health effect of BPA. Data were extracted on potential determinants of selective citation, such as study outcome, study design, sample size, journal impact factor, authority of the author, self-citation, and funding source. We applied random effect logistic regression to assess whether these determinants influence the likelihood of citation.

**Results:**

One hundred sixty-nine publications on BPA were identified, with 12,432 potential citation pathways of which 808 citations occurred. The network consisted of 63 cross-sectional studies, 34 cohort studies, 29 case-control studies, 35 narrative reviews, and 8 systematic reviews. Positive studies have a 1.5 times greater chance of being cited compared to negative studies. Additionally, the authority of the author and self-citation are consistently found to be positively associated with the likelihood of being cited. Overall, the network seems to be highly influenced by two highly cited publications, whereas 60 out of 169 publications received no citations.

**Conclusion:**

In the literature on BPA, citation is mostly driven by positive study outcome and author-related factors, such as high authority within the network. Interpreting the impact of these factors and the big influence of a few highly cited publications, it can be questioned to which extent the knowledge development in human literature on BPA is actually evidence-based.

**Electronic supplementary material:**

The online version of this article (10.1186/s41073-019-0065-7) contains supplementary material, which is available to authorized users.

## Introduction

Bisphenol A (BPA) is a chemical substance, which is used in plastics of, for example, food containers and can linings. It is considered a potential endocrine disruptor, as it might bind to estrogen receptors in the body and mimic estrogen’s function [1]. Most research of the potential harmful effects of BPA and its underlying mechanism has been conducted using in vitro studies or animal models [2]. In the in vitro setting, it was found that BPA can directly bind to androgen receptors and thereby block endogenous androgen action [3]. Because of its various uses, exposure to BPA in humans is widespread. Epidemiological studies linked exposure to BPA to a large variety of health outcomes, such as reproductive outcomes, metabolic diseases, behavioral outcomes, and intermediate health effects (e.g., DNA methylation and oxidative stress) [2, 4–6]. In 2012, the WHO concluded that the epidemiological evidence with respect to human health effects of BPA is limited and not coherent across the different health outcomes [7]. Additionally, the European Food Safety Authority has concluded that there is no health concern for humans at the expected level of intake [8]. In 2006, EFSA has set a tolerable daily intake (TDI) level of 0.05 mg per kilogram body weight per day [8]. This TDI is based on a no observed adverse effect level (NOAEL) determined in rodent studies and is also accepted in other countries, such as the USA and Japan [9]. BPA has not only been debated in the scientific community. It has also been a topic of extensive public debate, in which different stakeholders are involved such as industry and non-governmental organizations [10, 11]. The public discussion on the health risks of BPA, combined with the variety of BPA health effects, makes it a topic that is vulnerable to the distored use of evidence.. Especially when scientific evidence is the basis for decision-making processes, such as setting maximum levels of exposure, a complete and balanced view is crucial. Therefore, it is important to understand the knowledge development in this field of research.

Scientific knowledge development to a large extent is driven by citations. Due to the large and growing number of scientific publications in the biomedical domain and the limitation of the maximum number of references in many journals, it is often not feasible to refer to all available relevant literature [12]. In many cases, it is unclear on which grounds researchers decide to select the articles they cite. Selecting references based on their study results, usually meaning that positive studies are cited more often than negative studies, is called citation bias [13]. Citation bias has been studied in a variety of research areas, by using different methodologies and showing different results [14–17]. A recent systematic review has identified 52 studies on citation bias, from scientific disciplines in biomedical sciences, social sciences, and natural sciences. Twenty-nine of them found evidence for the existence of bias, whereas 12 studies found mixed results and 11 studies did not find evidence for the existence of citation bias [18]. Looking at the selection of references in a broader sense, authors might have different motives to select their references, which can take the form of justified (e.g., the methodological quality of a publication) or unjustified determinants (e.g., study outcome) of selective citation. Determinants that have been shown to be related to citation rate in multiple studies are sample size, study design, journal impact factors, and the number of references [19–21]. With regard to funding, it is often suggested that for-profit funding is less credible because only results that are preferred by the funder would be published [22]. A study by Kulkarni et al. showed that industry-funded studies that reported industry-favorable results were indeed associated with a higher annual citation count [23]. Factors that have been occasionally linked to citation count are the gender of the author, the number and type of affiliations included in a publication, the authors’ reputation, and whether the title of the publication includes its conclusion or not [19, 21]. It should be recognized that the effect of most determinants of selective citation will be located somewhere on the sliding scale between justified and unjustified determinants of citation with regard to their effect on knowledge development. It is the focus of the current study to identify which factors influence the development of knowledge by means of selective citations. The literature on BPA is used as a case study in this regard, which we chose because of its controversial nature and extensive public debate. Accordingly, we are not so much interested in the actual health effect of BPA and we will not make statements about this. For clarity reasons, we take the hypothesis that BPA has a harmful effect on human health as the starting point of this study.

The objective of this study is to assess the prevalence and determinants of selective citation in human studies on BPA in a quantitative manner.

## Methods

The design of this study was described in a study protocol, which was finalized and published online prior to the data collection (https://bit.ly/2kiDK4Z). The protocol is also available as Additional file 1 to this publication. The main steps of the citation network analysis will be described in the following paragraphs.

### Search strategy and article selection

All relevant publications were identified via Web of Science Core Collections, on 3 March 2017. For practical reasons, no other databases were searched, since only the Web of Science Core Collections has the possibility to download the reference lists of all publications. This information is needed to create the citation network and to perform the citation analysis. Identification of articles by checking the reference lists was not applied, since this would interfere with the research question. Checking reference lists would result in an overrepresentation of articles that are cited within the network, whereas articles that have been neglected by the network would still be missed. To prevent missing important publications, a broad search strategy was applied, namely (“Bisphenol A” OR “BPA”) AND (“Human*”). No limitations with regard to the health outcomes studied were applied.

**Fig. 1:**
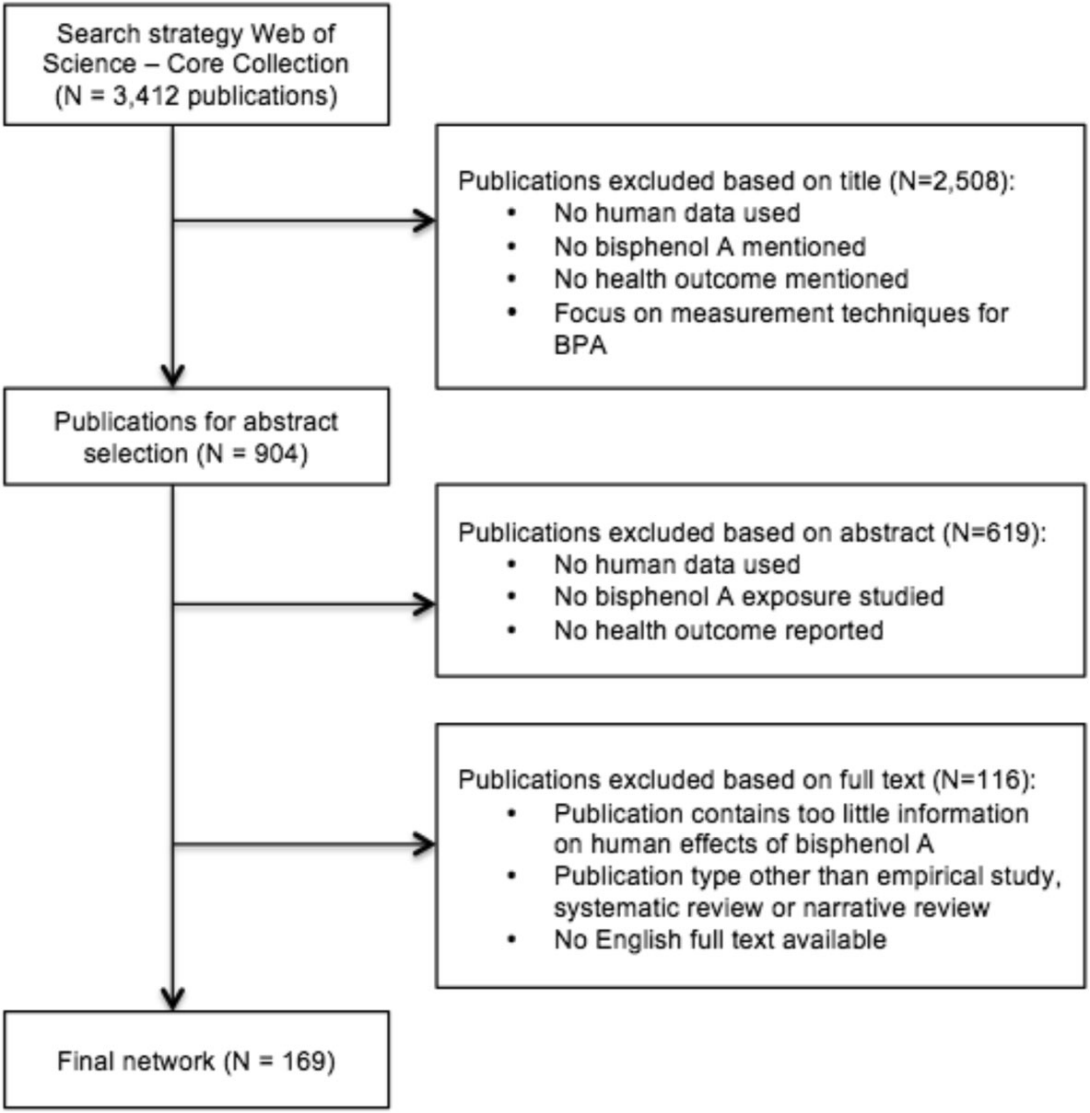
Flow diagram of the network selection process

The search strategy was very broad and not specific, in order to avoid missing relevant publication. This led to a large number of publications, namely 3412. The article selection was carried out in two phases. The first selection round was based on the publication title, to limit the number of publications. The second selection round included studying abstracts, figures, and tables, to finalize the network of human BPA studies. Many publications discussed BPA together with many other chemical compounds. By looking only at the abstract, it was not always clear to which extent the publication included information on BPA. By looking also at the figures and tables, we could make sure the publication contained sufficient information on BPA to be part of the network. The complete article selection was conducted individually by two researchers, MJEU and BD, followed by several consensus meetings. In case no consensus could be reached, a third researcher (GMHS) was asked to take a decision (Fig. 1).

### Data extraction

All publications in the network were scrutinized for a number of characteristics that may be potential determinants of citation (see Table 1). Data extraction was performed independently by MJEU and GMHS. In all cases, consensus was reached. Study outcome was scored in two ways. First, the data presented in the article were scored according to the reported statistical significance (statistically significant, not significant, or mixed). A publication was scored statistically significant when the primary study outcome reported a *p* value lower than 0.05. When multiple health outcomes were reported and the data showed *p* values both higher and lower than 0.05, a publication was considered mixed. Narrative reviews and systematic reviews without meta-analysis, which do not present new data, were not scored on their statistical significance. Secondly, the study outcome is scored by studying the authors’ conclusion of the publication. This can be either in line or not in line with the hypothesis that BPA has an adverse effect on human health. The health outcomes studied in the network were grouped into eight categories: reproductive outcomes, metabolic diseases, intermediate health parameters, hormone production, birth outcomes, behavioral outcomes, cancer, and others. The journal impact factor at the moment of publication was measured via the Web of Science*.*

**Table 1:** Characteristics of the bisphenol A network of 169 publications, 12,432 potential citation pathways and 808 realized citations

Variable	Categories	Number of publications	Number of potential citation pathways (% citations realized)
Study outcome			
Statistical significance	Yes	40	2800 (8%)
	No	36	2813 (5%)
	Mixed	47	3819 (9%)
	Not reported	46	2800 (8%)
Authors’ conclusion	In line with hypothesis	92	6826 (7%)
	Not in line with hypothesis	28	2114 (4%)
	Mixed	32	2658 (5%)
	Unclear	17	834 (9%)
Content-related determinants			
Health outcome	Reproductive outcomes	49	3853 (7%)
	Metabolic diseases	45	3218 (8%)
	Intermediate health factors	24	1594 (2%)
	Hormone production	18	1300 (8%)
	Birth outcomes	11	971 (6%)
	Behavioral outcomes	7	557 (3%)
	Cancer	4	286 (2%)
	Other	11	653 (6%)
Study design	Cohort study	34	2471 (7%)
	Cross-sectional study	63	5013 (9%)
	Case-control study	29	2019 (4%)
	Narrative review	35	2374 (4%)
	Systematic review	8	555 (5%)
Sample size*	< 168	42	3259 (6%)
	168–430	42	3154 (6%)
	> 430	43	3212 (10%)
Title of publication	Suggestive of conclusion	33	2327 (5%)
	Not suggestive of conclusion	136	10,105 (7%)
Not content-related determinants			
Number of affilations*	< 3	51	4241 (5%)
	3–5	59	3445 (7%)
	> 5	59	4746 (7%)
Journal impact factor*	< 2.85	60	4188 (5%)
	2.85–4.6	53	4210 (7%)
	> 4.6	56	4034 (8%)
Funding source	Not for profit	135	10,548 (7%)
	For profit	0	0 (0%)
	Both	4	369 (7%)
	Funding not reported	20	968 (3%)
	No funding applicable	10	547 (3%)
Number of references*	< 46	65	4152 (7%)
	46–58	47	4200 (8%)
	> 58	57	4080 (5%)
Corresponding author-related determinants			
Gender	Male	86	7359 (7%)
	Female	74	4472 (6%)
	Unknown	9	601 (3%)
Affiliation	University	136	10,403 (8%)
	Government	14	925 (6%)
	Industry	1	132 (1%)
	Other	18	1152 (7%)
Continent	America	73	6022 (8%)
	Asia	47	3612 (5%)
	Europe	42	2425 (4%)
	Australia	1	35 (3%)
	Africa	2	181 (2%)
	Middle East	4	157 (0%)

*For descriptive purposes, continuous variables have been transformed to categories based on tertiles

The determinant “authority of the corresponding author” was measured on the publication’s level and can vary over time. All co-authors of all publications received an “authority score,” which was the number of citations received within this BPA network, during each year that the network was active. The authority of each publication was determined by the co-author with the highest authority score. We hypothesized that authors with a high authority increased the credibility of a publication and therefore would lead to a higher likelihood of being cited. Self-citation was defined as the situation in which at least one author was listed on both the cited and the citing publication.

Continuous determinants that show a large range of values, which was often skewedly distributed, were divided into three categories, in order to reduce the variation and create more meaningful outcomes. This included the determinant’s sample size, journal impact factor, authority of the author, number of references, and number of affiliation. By making three categories for each determinant, each publication scores low, medium, or high in relation to the other publications in the network. Cutoff points between categories were based on tertiles, to make sure each category contained the same number of publications.

### Statistical analysis

Each publication in the network could take the role of the citing and the cited publication. We were solely interested in the effect of the characteristics of the cited publication on the likelihood of being cited, and therefore, the unit of analysis was the potential citation path. A potential citation path existed between one publication and every other publication in the network that was available online at the moment of submission. In the data set, each row represented a potential citation path followed by an indication whether the potential citation path had actually been realized or not and the characteristics of the cited publication of that citation path.

A single publication normally references multiple other publications, meaning that multiple citation pathways are leading to the same publication and are therefore not entirely independent. A multilevel approach was therefore required, in which the citation paths were nested under the citing publications. Random effect logistic regression was modeled to assess the effect of characteristics of the cited article on the likelihood of being cited.

First, univariate analyses were performed to test all potential determinants of citation, described in the previous paragraph, in the cited publication as a predictor for the likelihood of being cited. Second, all analyses were adjusted for study design, which was considered a proxy for study quality.

Additionally, we assessed whether concordance between the characteristics of the cited and citing publication was a determinant of citation. Via fixed effect logistic regression analysis, we tested whether concordance between the cited and citing publication determined the likelihood of citation. All statistical analyses were performed in Stata 13.

The outcomes of the logistic regression are reported as odds ratios. The odds ratio may overestimate the true relative risk in studies where the outcome is common [24]. In our network, the overall chance of being cited is 6.5% (808 actual citations of 12,432 potential citations). With this incidence, we consider “being cited” not very common, and consequently, the overestimation of the true relative risk will be small [24]. Ultimately, the odds ratio gives an accurate estimation of the direction of the effect; only the exact magnitude of the effect should be interpreted with some caution. For the sake of readability of the publication, we interpret these values as if they are relative risks and therefore, for instance, speak about “the likelihood of being cited for negative studies compared to positive studies.”

## Results

**Fig. 2:**
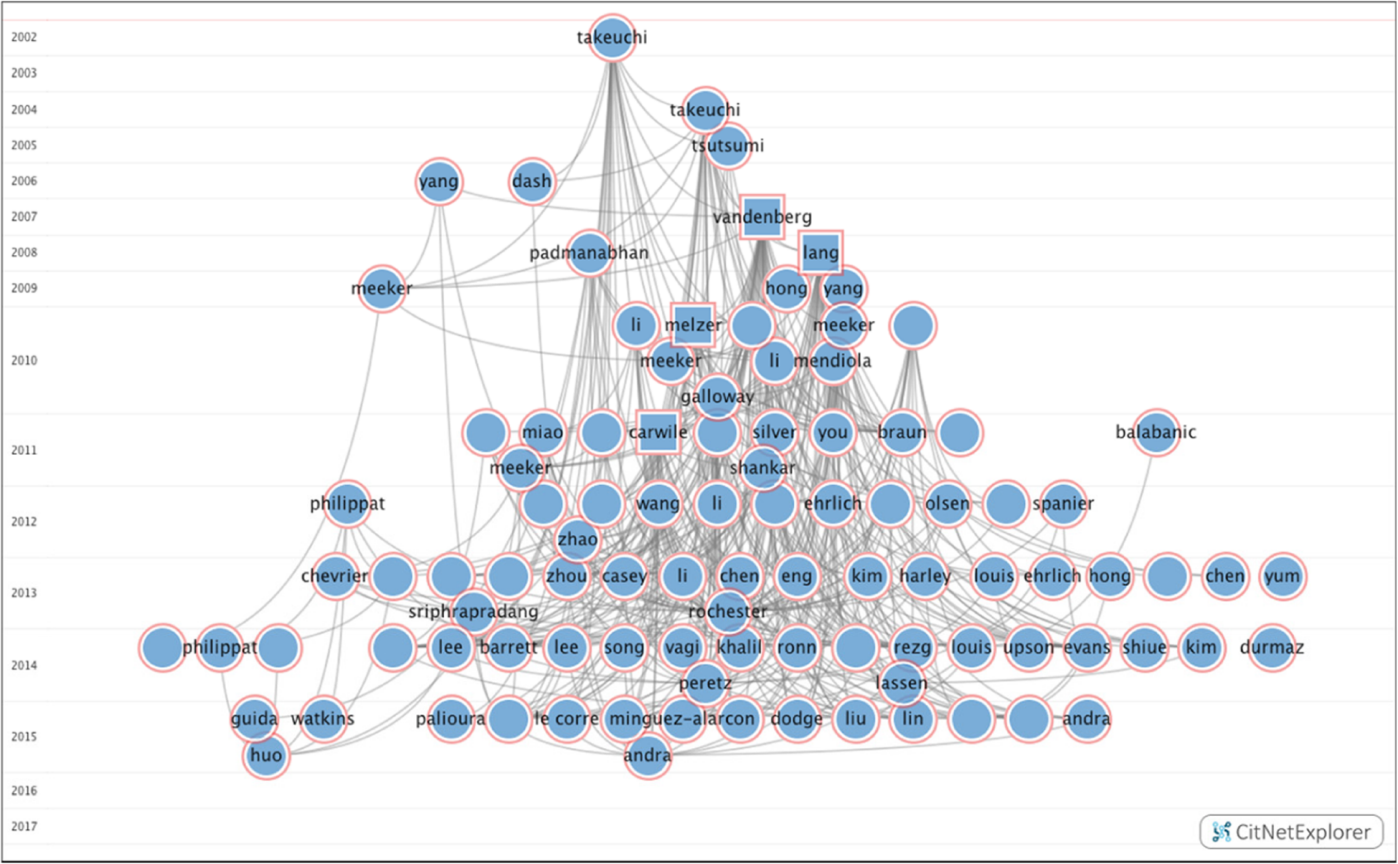
Visualization of the citation network, depicting the 100 most cited studies in the network. The *y*-axis depicts the timeline of publications, and the *x*-axis is solely for visualization purposes. Each circle and square is one publication, indicated by the name of the first author. The squares are the four most cited publications in the network. Each line indicates a performed citation from one publication to another

A network of 169 publications on human effects of BPA was identified, published between 2002 and the beginning of 2017. The publications are connected by 12,432 potential citations, of which only 808 citations were actually realized, making the likelihood of being cited in this network 6.5%. Figure 2 displays a visualization of a part of the citation network, including the 100 most cited publications. Each circle and square represents a publication, with the squares being highly cited publications with more than 30 citations each. The lines indicate a performed citation. On the *y*-axis, the timeline is indicated, ranging from 2002 to 2017. The *x*-axis is solely for visualization purposes.

### Publication characteristics

Table 1 describes the distribution of the potential determinants of citation over the publications in the network. BPA was most frequently studied in relation to reproductive outcomes (*N* = 49). The reproductive outcomes studied included, among others, polycystic ovary syndrome, miscarriage, sperm quality, and in vitro fertilization implementation failure. The study designs presented in this network were observational studies (experimental, cohort, cross-sectional, and case-control studies), systematic reviews, and narrative reviews. The network contains 126 publications that reported empirical data, which were summarized in 43 review publications. None of the systematic reviews included a meta-analysis. Since the network contained only one experimental study, this publication was classified as a cohort study. Looking at the evidence on adverse effects of BPA on human health, 40 publications reported statistically significant results, 36 publications reported non-significant results, and 47 publications reported mixed results. The authors of 92 publications concluded that there was a harmful effect of BPA on human health. A mixed or unclear conclusion was drawn in 49 publications, against 28 publications that concluded there was no harmful health effect of BPA. None of the studies was funded solely by for-profit organizations, which made it impossible to assess the effect of funding source as a determinant of citation. This underrepresentation of private parties in BPA research is also visible in the affiliation of the corresponding authors. Corresponding authors of 136 publications are affiliated with university whereas only one corresponding author is affiliated with industry.

### Citation pattern

Although the first human BPA studies were published in 2002, the majority of the literature is published from 2010 onwards. Nevertheless, it seems that some of the early studies attract a high number of citations. Two publications, a narrative review published in 2007 and a cross-sectional study published in 2008, received more than 50 citations [25, 26]. On the other hand, 60 publications in the network received 0 citations, which led to a very skewed distribution in the number of citations per publication. The median number of citations per publication was 1.

### Univariate and multivariate analyses (Table 2)

Study outcome, measured both as statistical significance and as author conclusion in line with the hypothesis that BPA is harmful to health, shows a significant positive association with the likelihood of citation. Significant and positive studies are approximately 1.5 times as likely to be cited compared to negative and non-significant studies, an effect that remains after the adjustment for study design. The concordance analysis showed that the study outcome was not likely to be concordant between the cited and citing publication (OR 1.06 (0.79–1.42), Additional file 2: Table S1).

**Table 2:** Univariate and multivariate analyses on potential determinants of the likelihood of being cited

Variable	Categories	Crude OR	Adjusted OR*
Study outcome			
Significance	Yes vs no	1.57 (1.28–1.92)	1.48 (1.21–1.80)
Authors’ conclusion	In line vs not in line with hypothesis	1.57 (1.29–1.92)	1.65 (1.34–2.03)
Content-related determinants			
Study design	Narrative review	1.00 (ref)	
	Cohort study	1.61 (1.26–2.07)	
	Cross-sectional study	2.00 (1.64–2.44)	
	Case-control study	1.08 (0.84–1.38)	
	Systematic review	1.36 (0.99–1.87)	
Sample size **	< 168	1 (ref)	1.00 (ref)
	168–430	1.04 (0.88–1.22)	1.00 (0.85–1.17)
	> 430	1.62 (1.27–2.05)	1.39 (1.12–1.74)
Title of publication	Suggestive title vs not suggestive title	1.25 (1.07–1.45)	1.16 (1.00–1.35)
Not content-related determinants			
Number of affiliations**	< 3	1.00 (ref)	1.00 (ref)
	3–5	1.46 (1.22–1.75)	1.27 (1.04–1.56)
	> 5	1.50 (1.24–1.82)	1.32 (1.06–1.65)
Journal Impact Factor**	< 2.8	1.00 (ref)	1.00 (Ref)
	2.8–4.6	1.21 (1.07–1.36)	1.08 (0.96–1.22)
	> 4.6	1.41 (1.22–1.63)	1.22 (1.06–1.41)
Funding source	For-profit vs not-for-profit ***	NA	NA
	Not reported vs reported	0.41 (0.31–0.55)	0.74 (0.43–1.28)
Number of references**	< 46	1.00(ref)	1.00 (ref)
	46–58	1.24 (1.08–1.42)	1.10 (0.95–1.26)
	> 58	0.75 (0.63–0.89)	0.78 (0.65–0.92)
Author-related determinants			
Gender of corresponding author	Male vs Female	1.00 (0.89–1.11)	0.97 (0.86–1.09)
Affiliation of corresponding author	Private vs public sector	0.94 (0.75–1.17)	1.31 (0.57–3.01)
Authority of the authors**	< 3	1.00 (ref)	1.00 (ref)
	3–26	2.21 (1.84–2.66)	2.16 (1.78–2.63)
	> 26	3.20 (2.59–3.96)	3.32 (2.64–4.18)
Self-citation	Yes vs no	5.14 (3.88–6.81)	5.16 (3.81–6.99)

*Adjusted model is adjusted for study design

**Continuous variables were categorized based on tertiles

***None of the publications was funded solely by for-profit organizations, therefore this analysis was not possible

Contrary to our expectation, systematic reviews were not more frequently cited than narrative reviews in the full network. Sample size, number of affiliations, and journal impact factor showed a moderate positive association with the likelihood of being cited, with ORs between 1 and 2. These effects could partly be explained by study design. The type of affiliation of the corresponding author, gender of the corresponding author, report of funding, and number of references showed no association with citation. Authority of the author and self-citation was found to have the strongest association with the likelihood of being cited. High authority, which was measured by a combination of the number of publications and the number of earlier citations in this field, increased the likelihood of citation by approximately three times. Authors were five times more likely to cite their own work compared to that of others.

### Sensitivity analysis

Knowing that the number of citations per publication is very skewedly distributed, we tested to which extent the results are driven by the two highly cited studies. As a data-driven, post hoc analysis, we excluded these two studies, which received more than 50 citations (Table 3). The significant effects that were found for the sample size and the number of affiliations in the overall network disappeared. This can be explained by the fact that one of the highly cited studies was a cross-sectional study with a large sample size of 1455 participants [26]. The other highly cited study was a narrative review, which means the study had no specified sample size [25]. Both studies were performed by relatively large research groups of five and six affiliations, respectively. In this sensitivity analysis, the study outcome, journal impact factor, authority of the author, and self-citation remained significantly associated with citation.

**Table 3:** Sensitivity analysis: crude and adjusted odds ratios for the chance of being cited, excluding two highly cited publications (> 50 citations)

Variable	Categories	Crude OR	Adjusted OR*
Significance	Yes vs no	1.57 (1.28–1.92)	1.48 (1.21–1.80)
Authors’ conclusion	In line vs not in line with hypothesis	1.45 (1.18–1.77)	1.53 (1.25–1.87)
Study design	Narrative review	1.00 (ref)	
	Cross-sectional study	4.44 (3.26–6.04)	
	Case-control study	2.61 (1.88–3.62)	
	Cohort study	3.93 (2.82–5.46)	
	Systematic review	3.28 (2.22–4.85)	
Sample size**	< 168	1.00 (ref)	1.00 (ref)
	168–430	1.03 (0.87–1.21)	0.98 (0.84–1.16)
	> 430	1.40 (1.09–1.79)	1.22 (0.97–1.54)
Title of publication	Conclusive title vs non-conclusive title	1.07 (0.91–1.25)	0.96 (0.82–1.12)
Number of afilliations**	< 3	1.00 (ref)	1.00 (ref)
	3–5	1.46 (1.21–1.77)	1.06 (0.86–1.30)
	> 5	1.09 (0.86–1.38)	0.70 (0.55–0.90)
Journal impact factor**	< 2.8	1.00 (ref)	1.00 (ref)
	2.8–4.6	1.62 (1.41–1.87)	1.38 (1.19–1.60)
	> 4.6	1.59 (1.33–1.89)	1.43 (1.20–1.70)
Funding source	Not reported vs reported	0.46 (0.34–0.63)	0.83 (0.62–1.13)
Number of references**	< 46	1.00 (ref)	1.00 (ref)
	46–58	1.07 (0.92–1.24)	0.96 (0.82–1.12)
	> 58	0.51 (0.42–0.61)	0.56 (0.46–0.69)
Gender of corresponding author	Male vs female	1.14 (1.01–1.28)	1.20 (1.05–1.37)
Affiliation of corresponding author	Private vs public sector	1.07 (0.86–1.35)	1.08 (0.85–1.38)
Authority of the authors**	< 3	1.00 (ref)	1.00 (ref)
	3–26	2.26 (1.85–2.76)	2.09 (1.68–2.60)
	> 26	2.75 (2.18–3.47)	2.69 (2.08–3.48)
Self-citation	Yes vs no	5.46 (4.09–7.28)	5.05 (3.75–6.81)

*Adjusted model is adjusted for study design

**Continuous variables were categorized based on tertiles

## Discussion

With this citation network analysis, we aimed to quantify the occurrence of citation bias in the human BPA literature and the determinants that influence the citation behavior in this field. Based on the finding that positive studies have an approximately 1.5 higher likelihood of being cited compared to negative studies, we conclude that citation bias is present in the BPA literature, although its magnitude might be limited. This effect was not confounded by study design and remained after excluding the most highly cited studies. Also, based on the results from the concordance analysis, citation bias does not appear to be influenced by the study outcome of the citing publication.

These results are in line with the findings of a recent meta-analysis on citation bias in various scientific fields, of which most were biomedical [18]. This systematic review and meta-analysis showed that citation bias is prevalent throughout multiple biomedical research fields and that significant findings lead to an approximately 1.5 times higher chance of citation compared to non-significant findings [18]. This was a pooled effect over a variety of disciplines, such as Alzheimer’s disease, coronary heart disease, and psychiatry [27–29]. Also, the finding that the authors’ conclusion has a stronger effect on citation than the significance level of the data was confirmed by previous research in this meta-analysis [18].

The second aim of this study was to assess the effect of other potential determinants of citation. In the complete network of 169 publications, sample size, journal impact factor, the number of affiliations involved, the authority of the author, and self-citation were found to affect the likelihood of being cited. This was in line with our expectations, based on previous research in different research areas [30, 31]. However, after the exclusion of the two publications with the highest number of citations, only the journal impact factor, authority of the authors, and occurrence of self-citation appeared to be stable determinants of citation in the BPA literature. Different than the study outcome influencing the likelihood of citation, the occurrence of self-citation is not necessarily leading to biased knowledge development. To some extent, self-citation is inevitable since academics are working to expand on their previous work [32]. Of course, it might lead to selective overrepresentation of certain results and their interpretation and thereby skew knowledge development [32]. Additionally, self-citation might be a way for authors to promote their own vision, which might lead to an author-based instead of evidence-based knowledge development. Before drawing conclusions on the possible effect of self-citation on knowledge development, we should keep in mind that self-citations can be used in different ways, apart from promoting certain results and substantiating an argument, authors refer to their own work to introduce a method that was described earlier or to explain the relevance of their research topic [33]. Based on the current research, we could not conclude whether the amount of self-citation leads to a biased knowledge development in the BPA literature, since we did not asses in which paragraph of the publication self-citations were used.

In addition to the citation bias found, we should be aware that a large proportion of the literature seems to be completely ignored. More than one third of the publications receive zero citations, and even though these are both positive and negative publications, it means that part of the evidence is being left out of the picture and researchers are not appreciated for their work. Looking at the distribution of the number of citations per publication over time, it seems that the highly cited publications are early publications in the field. With the growing amount of literature, the chance of not being cited at all seems to increase. Although it is logical and acceptable that founding publications are often mentioned to describe the research field, we should be aware that the more recent evidence is less often referred to. Especially because BPA is a research field that is highly debated in risk assessment and risk management procedures, it is important to have a complete overview of all available evidence. The finding that a big part of literature is not valued in terms of citations has also been found in other research fields [34–36]. For example, Robinson and Goodman showed that in the field of clinical trials, only a quarter of available trials got cited in the development of a new trial. Also, the number of trials that were cited did not increase with a bigger number of available trials [34]. This gives support to the idea that an abundance of literature leads to reduced visibility for individual publications, potentially leading to research waste and misinterpretation of the literature in decision-making processes.

If we look, on the other hand, at the highly cited publications, it is remarkable that these studies have a narrative review and a cross-sectional study design, both of which are study designs that are typically not very highly valued. Although we did not look into the content of the publications in this study, we should be aware that both studies do not give a complete overview of the literature, as a systematic review would do, and thereby improve the chance of skewed knowledge development.

One of the limitations of the current study is that the search strategy was only applied to the Web of Science Core Collection, making it quite possible that some relevant publications have been missed. The search was limited to this database because Web of Science is the only database that has the option to download the publications together with their reference lists. This information was necessary to set up the database and perform the statistical analysis. Nevertheless, we have no reason to believe that the identified determinants of selective citation would be different if literature from other sources would have been included in the network. A related limitation was the fact that we did not check reference lists for missing publications. This might have led to missing relevant publications. However, we believe that checking of reference lists might have interfered with our research question. Checking of reference lists would only identify publications that were actually cited within the network, while still missing relevant publications that did not receive any citations.

## Conclusion

Concluding, we found proof that citation bias is present in the human literature on BPA. Publications that concluded a harmful health effect of BPA are 1.5 times more likely to be cited compared to negative publications. The association between other determinants and the chance of being cited is found to be hard to quantify since our analysis was highly influenced by a low number of highly cited publications. Nevertheless, journal impact factor and author-related factors such as author’s authority and self-citation show a consistent positive association with the chance of being cited. With these findings, we could conclude that the development of the available knowledge on BPA seems to be mostly driven by authority-related factors, instead of by the best available evidence.

## Additional files

Additional file 1: Research protocol. (DOCX 436 kb)

Additional file 2: **Table S1**. Effect of concordance between the cited and citing article on the likelihood of being cited. Reference list bisphenol A network. (DOCX 80 kb)

## Abbreviations

BPA: Bisphenol A
OR: Odds ratio
