# Supplementary material for: Selective citation in scientific literature on the human health effects of bisphenol A
Source: Res Integr Peer Rev. 2019 Mar 29;4:6. doi: 10.1186/s41073-019-0065-7 (PMC6440006; doi:10.1186/s41073-019-0065-7)
Supplement: Supplementary file 1 — Research protocol. (DOCX 436 kb) [file 41073_2019_65_MOESM1_ESM.docx]

**Study protocol: A citation network analysis on epidemiological studies concerning bisphenol A**

Miriam J.E. Urlings, Bram Duyx, Gerard M. Swaen, Lex M. Bouter, Maurice P. Zeegers

MJEU: CAPHRI School for Public Health and Primary Care, Maastricht University, Maastricht

BD: CAPHRI School for Public Health and Primary Care, Maastricht University, Maastricht

GMS: CAPHRI School for Public Health and Primary Care, Maastricht University, Maastricht

LMB: Department of Epidemiology and Biostatistics, VU University Medical Center, Amsterdam

MPZ: CAPHRI School for Public Health and Primary Care, Maastricht University, Maastricht

This study is sponsored by NUTRIM, Maastricht University and CEFIC/LRI. The sponsors had no role in the development of the research protocol.

**Background**

Citations are often regarded as an indicator of scientific quality([1](#_ENREF_1)). Numbers of citations are sometimes used to assess the quality and societal impact of a paper([2](#_ENREF_2)). The rationale behind this is that high quality work will lead to more citations by peer scientists compared to low quality work([1](#_ENREF_1)). However, it can be questioned whether it is scientific merit, that drives the number of citations, or other factors such as study outcome or language.

Due to the large and growing number of scientific articles in the biomedical domain and limitation of the maximum number of references in many journals, it is often not feasible to cite all available literature. Ideally, a representative sample of available literature is cited. However, it is unclear on which ground researchers decide to select the articles they reference and whether these references always are a representative sample of the available data. If an unbalanced overview of the available literature is given, e.g. by citing more often articles with a specific study outcome, this is called citation bias. Citation bias results in disproportionate attention for specific part of the literature, while other data are being systematically neglected. Because disproportionate attention goes to a specific part of the available evidence, a distorted consensus can develop.

Citation bias is one example of a Questionable Research Practice (QRP). QRP’s are subtle misbehaviours in science, and cover the grey area between responsible research conduct and serious research misconduct. Although QRP is more subtle than scientific misconduct such as fabrication, falsification and plagiarism, it is a serious threat for the development of scientific knowledge([3](#_ENREF_3)). To enhance and ensure integrity in academic research, empirical citation analyses are required to obtain insight in the occurence and causes of citation bias as part of QRP. Citation analysis can be performed in several manners. Greenberg (2009) performed a network analysis based on a specific scientific claim. After identifying all literature (primary data articles, model studies and reviews) on this statement, he compared the number of citations to critical primary data publications with the number of citations to supportive primary data publications. From this network analysis it was concluded that 94% of the citations was assigned to the supportive data([4](#_ENREF_4)). Apart from a claim-specific network, a network analysis can be based on a specific journal or time period. Fanelli (2013) performed a citation analysis on hypothesis-testing studies from several research domains,

published between 2000 and 2007. It was concluded that positive studies received on average 32% more citations than negative studies([5](#_ENREF_5)).

In the proposed study, a claim specific network analysis will be performed. The citation pathway will be taken as the unit of analysis. The occupied citation pathways will be compared to all potential citation pathways, at the time of publication. This is important, in order to control for the complete number of potential citations at each moment in time and the proportion of positive and negative studies available. In contrast to Greenberg’s approach, other determinants on top of study outcome are taken into account to test if the relationship between study outcome and citation can be explained by other determinants of citation.

Authors might have different motivations to select their citations; these motivations can take the form of justified (e.g. the methodological quality of a paper) or unjustified (e.g. study outcome) determinants of selective citation. For the present study we have identified one unjustified determinant of selective citation: study outcome. If citation selection is motivated by study outcome, a distorted view of the available knowledge will be presented, resulting in citation bias. On the other hand, there might be more justified determinants of selective citation, such as research quality. It is expected, that justified determinants do not negatively impact the knowledge and consensus development. Nevertheless, it should be recognized that most potential determinants will be located somewhere on the sliding scale between justified and unjustified determinants.

The objective of this study is to quantify the prevalence of citation bias in a specific research field and to get insight in justified and unjustified factors that influence the selection of citations.

**Bisphenol A**

As an example topic, the determinants of selective citation will be studied in the epidemiological literature concerning bisphenol A. Biphenol A (BPA) is a chemical substance, used in plastics in food containers and can linings. It is considered a potential endocrine disruptor, as it might connect to estrogen receptors in the body and mimic the estrogen’s function([6](#_ENREF_6)). Most research of the potential harmful effects of BPA and its underlying mechanism has been conducted via in vitro studies or in vivo animal models([7](#_ENREF_7)).

Furthermore, epidemiological studies have focused studied the effect of BPA on a lage variety of health outcomes in humans. The health outcomes under study range from fertility related outcomes, to metabolic diseases and behavioral outcomes([7](#_ENREF_7), [8](#_ENREF_8)). The WHO concluded in 2012 that epidemiological evidence with respect to human health effects of bisphenol A is limited and not coherent over the different health outcomes([9](#_ENREF_9)). With this citation network analysis we aim to map all published epidemiological research on health effects of BPA and assess which factors drive selective citation in this area of research.

Our main research question is: Which determinants influence the likelihood of being cited in the epidemiological literature on human health effects of bisphenol A?

**Methods**

The relevant scientific literature will be systematically reviewed and visualised in a citation network. The network will include empirical papers (cross-sectional studies, cohort studies, intervention studies) and synthesis papers (reviews, meta-analyses, editorials). This is an important distinction, as empirical papers generate their own data to base their statements on, whereas synthesis articles use other publications to make a summary of the literature.

**Information sources**

All relevant publications will be identified via Web of Science – Core Collections. Identification of articles by reference checking will not be applied, since this would interfere with the research question. This would result in an overrepresentation of articles that are cited within the network, whereas articles that have been neglected by the network will not be identified. The citation network of all relevant articles will be visualised by using CitNetExplorer. In CitNetExplorer, the papers and corresponding citations between each paper will be visualised in chronological order. This can be useful as a first step to see general citation patterns.

**Search strategy**

First a broad search strategy will be conducted in Web of Science, to prevent missing out on important publications. Concerning the determinant under study, the search terms “Bisphenol A” OR “BPA” will be used. With regard to health outcome, this study will not apply any limitations. All epidemiological studies assessing the effect of bisphenol A on human health will be included. Therefore, no search terms relating to the study outcome are used, but a filter for “human” was applied on the search, since we are only interested in human studies.

**Article selection process**

Article selection will be carried out in two phases. The first selection round is aimed at making a broad selection of articles belonging to the network, based on their title. In a second selection round abstracts, figures and tables will be studied, to make sure only human epidemiological studies on health effects of bisphenol A will be included. This methodology has been chosen to minimize the risk of overlooking relevant articles. In Table 1, the in- and exclusion criteria are depicted. Both selection rounds will be conducted by two researchers, MJEU and BD, followed by consensus meetings. In case no consensus can be reached, a third researcher (GS) will be involved to take a decision.

*Table 1: In- and exclusion criteria that will be applied to identify all relevant epidemiological publications on human health effects of bisphenol A*

| Inclusion criteria | Exclusion criteria |
| --- | --- |
| Epidemiological studies on human subjects studying the association between bisphenol A and any health effect | In vitro or animal studies on the potential health effects of bisphenol A |
|  | Ecological studies on exposure rates of bisphenol A |
|  | Policy documents on the regulation of availability of bisphenol A in the environment |

**Article characteristics**

MJEU and BD will independently score the article characteristics that might be potential determinants of citation. The potential determinants of selective citation are article characteristics that can be present both in the cited and the citing article. A list of potential determinants of citation has been identified via literature search and consultations with researchers. They include study outcome, study design, sample size, number of affiliations involved, authority of authors, journal impact factor, funding source, continent, affiliation of the corresponding author, gender of the corresponding author, number of references, use of self-citations and the title of the publication. In addition, the concordance between the characteristics of the cited and citing article will be assessed. Where possible, article characteristics will be marked as ‘justified’ or ‘unjustified’ determinant of selective citation. This refers to the ability of the determinant of selective citation to bias the knowledge dissemination. If citations are selected based on justified determinants, this will not lead to skewed knowledge development, whereas bias might occur if unjustified determinants are driving citation. However, there is a grey area between justified and unjustified determinants. In this grey area, most article characteristics will be located as determinant of selective citation.

**Study outcome**

It is suspected that study outcome is a strong predictor of citation in several research fields([10](#_ENREF_10)). Study outcome will be operationalized in two ways. First we will score if the publication reports statistical significant results concerning the effect of bisphenol A exposure on human health. Second, we will score the publication’s vision towards the hypothesis that bisphenol A exposure has a negative effect on human health. Publications can be in line, not in line or neutral with regard to this hypothesis. In both operationalisations, ‘human health’ can be defined as any published health outcome. In case more than one health outcomes are discussed, the publication will be scored as ‘multiple outcomes’. If more than 50% of the discussed diseases report a negative effect on health, this will be scored as a study being in line with the hypothesis. If less than 50% of the discussed study outcomes report a negative effect on health, the publication is scored as not in line with the hypothesis.

These determinants will be measured and analysed at the level of the cited article. Also the study outcome of the citing article will be measured, to assess the concordance between the study outcome of the citing and cited article. Selecting citations based on the study outcome would be an unjustified determinant of selective citation and could result in misrepresentation of the available evidence.

**Health outcome**

Bisphenol A has been studied in relation to a variety of health outcomes, such as obesity, fertility related diseases and cancer ([7](#_ENREF_7), [8](#_ENREF_8), [11](#_ENREF_11)). The type of health outcome will be scored for each publication. This variable will be analysed in direct relation to citation, to assess if some diseases receive more citations than others in the complete BPA network. Additionally, the variable health outcome will be analysed in the concordance analysis, to determine if authors mostly cite publications with a similar health outcome. In this way we can assess if the epidemiological literature on BPA is one large literature network, or if certain health outcomes form their own subnetwork within the BPA literature and only cite to each other.

**Study design**

Study designs can roughly be divided into primary data papers and synthesis papers. The primary data papers are grouped in three specific study designs: cross sectional studies, cohort studies and intervention studies. Synthesis publications are grouped in systematic reviews without meta-analyses, systematic reviews including meta-analysis, narrative reviews and editorials. The difference between systematic and narrative review will be scored based on the presence or absence of a systematic search strategy described in the publication or supplemental materials. The division between primary data publications and synthesis publications is important, since we expect that the effect of selective citation in primary data articles have less impact than selective citation in synthesis articles. After all, primary data articles are based on their own data and draw conclusions based on their own findings, whereas synthesis articles draw conclusions based on their citations. In addition, systematic reviews and meta-analyses have the responsibility to summarise all available evidence in a specific domain and therefore should not cite selectively.

The study design of the citing and the cited article will both be documented. The effect of the study design of the cited article on citation will be analysed. Next, the effect of the concordance between the study design of the citing and cited article on citation will be analysed.

**Sample size**

The number of study subjects in each publication will be documented. The sample size is a measure of the robustness of the statistical findings and therefore, a larger sample size could be a justified determinant for selective citation. Sample size will be decoded into a categorical variable, based on its tertiles. In the analysis, the impact of the sample size of the cited article on citation will be tested. In addition, the sample size of the citing article will be measured in order to test the concordance between the study design of the citing and cited article and its effect on citation.

**Number of affiliations involved**

The number of affiliations that contributed to the cited article will be reported. It is hypothesized that a higher number of affiliations, a consortium, increases the credibility of an article and therefore, it receives more citations. In addition, a higher number of affiliations

involve a bigger network of scientists, which might be more likely to cite to their peers. In the analysis, the number of affiliation will be recoded into a categorical variable, based on its tertiles. The number of affiliations of the citing article will also be documented, in order to assess the concordance between the number of authors of the cited and citing article.

**Journal Impact factor**

The journal impact factor has often been suggested to be a measure of scientific quality and scientific impact, as it refers to the number of citations a journal has received in the previous two years. Therefore, article published in a journal with a high impact factor are likely to be perceived as being of high scientific quality, leading to more citations. The other way around, it might be helpful to cite to articles published in high impact factor journals in getting published in a high impact factor journal yourself. In the analysis, the effect of the impact factor of the cited article on citation will be tested and the concordance between the impact factor of the cited and citing publication will be analysed for its effect on citation.

**Funding source**

The funding of publications can be come from for-profit or not-for-profit organizations. Possibly, the funding source has an influence on the reporting of an article. Literature suggests that therapeutic trials funded by industry are more likely to report positive study outcomes compared to trials funded by public institutions and that the trials that received the most citations are funded by industry([12](#_ENREF_12)). It will be analysed if the funding source of the cited article has any effect on whether citation pathways are being used or not. In addition, the concordance between the funding source of the cited and citing article will be measured and its effect on citation will be analysed.

**Country**

It might be possible that authors mainly cite authors from a specific region or culture, preferably their own. Several articles have suggested that, especially American authors, tend to cite their colleagues from their own continent([13](#_ENREF_13)). In assessing the effect of country on citation, the country of the author of the cited article will be taken into account. In addition, we will check if there is concordance between the continent of the author of the citing and cited author and if this influences the chance of citation. In case more countries are represented on an article, this will be coded as ‘ multiple countries’.

**Affiliation**

The affiliation of the corresponding author will measured and will be categorized as private or public institution. Since the affiliation of a publication’s author is not always coherent with the funder of the publication, this will be analysed separately. Affiliations related to governments (e.g. universities, university hospitals or governmental research institutions) will be scored as public institutions. Private institutions include industry and privately funded research centres. Scoring the affiliation will be done based on the first affiliation of the first author. The impact of the affiliation of the cited author on citation will be analysed. In addition, the concordance between the affiliation of the cited and citing author will be tested and its effect on citation will be assessed.

**Gender**

There are some studies suggesting that publications authored by males are more likely to receive citations compared to publications with a female first author. However, data on the relationship between gender of the author and the number of citations are inconsistent and might differ between scientific fields ([14](#_ENREF_14)). The gender of the corresponding author of the cited and citing article will be documented. The gender of the author of the cited article will be analysed as determinant of citation. Also the concordance between the gender of the cited and citing author and its effect on citation will be tested.

**Authority of the author**

An article can attract a greater number of citations because of the authority of one or more of the authors. When one of the authors is an authority in the field, the article might be more easily available and other scientists in the field might give more credits to this article. The authority of an author will be operationalized as the number of citations he or she has received within the network, at each moment one of his or her papers is cited by one of the citing papers. The number of citations received within the network will be documented for each author at the moment of a new possible citation. The author with the highest number of citations at that time, will be used in the analyses as ‘the authority’. The effect of authority the author of the cited article on citation will be analysed. Also the effect of concordance between the authority of the authors of the cited and citing article on citation will be assessed.

**Number of references**

The number of references of the cited study will be included in this study as a determinant for receiving future citations. It might be the case that a high number of references increases the perceived quality of the publication. In addition, the effect of the concordance between the number of references of the citing and cited article will be analyzed.

**Self-citation**

Researchers are likely to cite their own work and the work of people they have been working with in the past. For this project, self-citation is defined as the situation in which at least one of the authors is authoring both the citing and the cited article. Since there is no self-citation within the citing and cited article, only the concordance between the cited and citing article will be tested.

**Title of the publication**

A first impression of an article is often based on the title. The title can clearly suggest whether or not an effect is reported in the article. On the other hand, a title can be more objective, not giving a suggestion on the analytic conclusion of the article. It is suspected that a title suggesting whether an effect is concluded would receive more citations compared to a neutral title. The title of the cited article will be scored as suggesting whether or not an association is found vs. no suggestion on the analytic conclusion. As such it will be analysed as a determinant of citation, as well as the concordance between the title of the cited and citing article.

*Table 3. Article characteristics and subsequent operationalization*

| Characteristic | **Operationalization** |
| --- | --- |
| Statistical significance | 0 = not significant study outcome (95% CI includes the neutral value of the effect parameter)  1 = significant study outcome (95% CI does not include the neutral value of the effect paramater)  2 = No significance reported  3 = Mixed results |
| In line with hypothesis | 0 = Not in line with the hypothesis that bisphenol A exposure has a negative effect on human health  1 = In line with the hypothesis that bisphenol A exposure has a negative effect on human health  2 = Unclear outcome |
| Study design | 1 = Cohort study  2 = Cross-sectional study  3 = Intervention study  4 = Review paper  5 = Other synthesis paper (e.g. editorials) |
| Sample size | Number |
| Number of affiliations | Number |
| Journal Impact Factor | Journal impact factor at the time of publication |
| Funding source | 0 = For profit organisation  1 = Non-profit organisation  2 = Both  3 = Not reported  4 = Not applicable |
| Country | Free text |
| Affiliation corresponding author | 0 = University  1 = Government  2 = Industry  3 = Other |
| Gender corresponding author | 0 = Male  1 = Female  2 = Unknown |
| Number of references of the cited article | Number |
| Authority of author | Number of citations received within the network, at the time of citation |
| Self-citation | 0 = No  1 = Yes |
| Title of the publication | 0 = Title gives no suggestion on the analytic conclusion of the article  1 = Title suggests whether or not an association between determinant and health outcome is concluded |

**Statistical analysis**

The unit of analysis will be the citation pathway (either being used or not) between any pair of articles in the network. All potential citation pathways are defined as the links between articles that have been published at least 1 year after each other. This limit of one year has been chosen because it can take around two years for the citing article to be written, reviewed and published, while the reference list may not alter much in this period. The outcome is defined as whether a particular citation pathway is utilized or not. This will be regressed against the previously described characteristics of the cited article via random effects logistic regression in which the correlation structure of the citation network is taking into account. In table 4 the data extraction form with all characteristics that will be measured in each publication are depicted.

*Table 4. Measured variables – possible determinants of citation*

| Article number | Statistical significance BPA | In line with hypothesis BPA | Study design | Sample size | Number of co-authors | Journal Impact Factor | Country | Affiliation | Gender | No. of references | Authority of authors | Effect reported in title | Self-citation |
| --- | --- | --- | --- | --- | --- | --- | --- | --- | --- | --- | --- | --- | --- |
| 1 |  |  |  |  |  |  |  |  |  |  |  |  |  |
| 2 |  |  |  |  |  |  |  |  |  |  |  |  |  |
| 3 |  |  |  |  |  |  |  |  |  |  |  |  |  |

**Data set**

In order to perform the statistical analysis, a data set needs to be set up. The data set will be designed based on the possible citation pathways. To maintain a proper overview, all articles are displayed in a citation matrix, as shown in figure 1. In the citation network, each article can have the role of citing and cited article. Nevertheless, citation pathways have only one dimension, namely directed towards the past. To assure a study can be cited, we need to take into account the publication period for each publication to become available, since no citations will be added after a manuscript is submitted to a journal. This publication period is calculated by subtracting the publication date of the ‘cited’ article (when the article becomes accessible for other scientists) from the submission date of the ‘citing’ article (after which hardly any changes will be made to the article).

Note: the terms ‘citing’ and ‘cited’ refer to the *potentially* citing and cited articles.

| Citing article | Cited article | | | | | | | | | | |
| --- | --- | --- | --- | --- | --- | --- | --- | --- | --- | --- | --- |
|  |  | 1 | 2 | 3 | 4 | 5 | 6 | 7 | 8 | 9 | 10 |
|  | 1 | - |  |  |  |  |  |  |  |  |  |
|  | 2 | - | - |  |  |  |  |  |  |  |  |
|  | 3 | - | - | - |  |  |  |  |  |  |  |
|  | 4 | - | - | - | - |  |  |  |  |  |  |
|  | 5 | - | - | - | - | - |  |  |  |  |  |
|  | 6 | - | - | - | - | - | - |  |  |  |  |
|  | 7 | - | - | - | - | - | - | - |  |  |  |
|  | 8 | - | - | - | - | - | - | - | - |  |  |
|  | 9 | - | - | - | - | - | - | - | - | - |  |
|  | 10 | - | - | - | - | - | - | - | - | - | - |

*Figure 1. Citation matrix of all the possible citation relations.*

The data set is displayed in table 5. Each row of the data set will represent a potential citation pathway. The third column will state if the citation pathway is utilized or not, this information will be distracted from the citation matrix. Thereafter, all the characteristics of the cited and the citing article corresponding to that row, will be documented. The characteristics of the citing article need to be documented in order to assess the concordance of each characteristic between the citing and cited article.

*Table 5. Data set*

| Cited article number |  | Citing article number |  | Occupation citation path |  | Measured variables cited article |  | Measured variables citing article |
| --- | --- | --- | --- | --- | --- | --- | --- | --- |
|  |  |  |  |  |  |  |  |  |
|  |  |  |  |  |  |  |  |  |
|  |  |  |  |  |  |  |  |  |

**Visualization citation network**

Before starting the statistical analysis, we make a visualization of the citation network in the computer program CitNetExplorer. In figure 2 an example of a citation network is shown. The dots in the picture represent the articles in the network and the lines are the citations towards articles in the network. On the y-axis the publication year is indicated, so the development over the years can be followed.

**
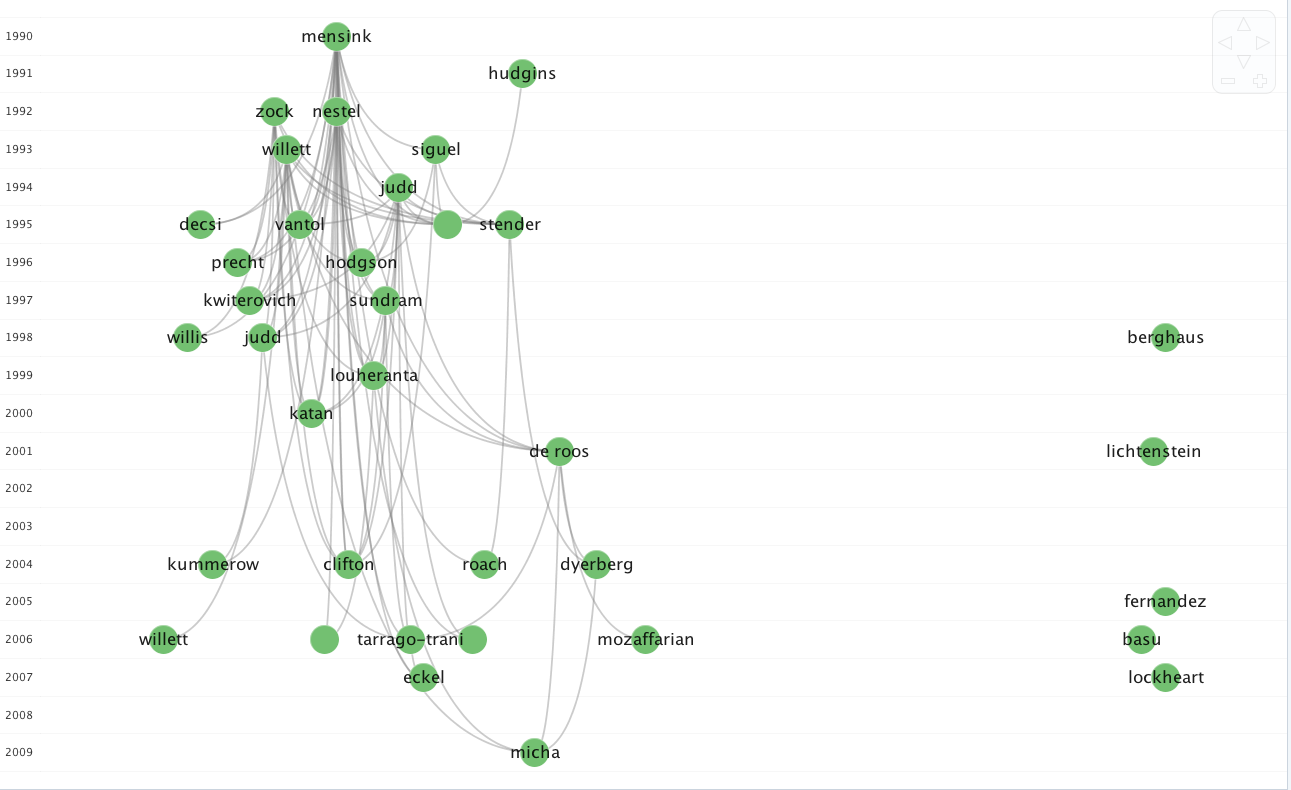
**

*Figure 2. Example of citation network visualized in CitNetExplorer*

**Random effect logistic regression**

Univariate analyses will be performed for each determinant of the cited article to see if there is an effect on the likelihood of being cited. When investigating the potential impact of the characteristics of the cited articles on being cited, we will perform a random effect logistic regression analysis in which clustering within the citing articles is taken into account.

Multivariate analysis will adjust each determinant for the variables study design, sample size and presence of an a priori hypothesis. These variables are used as a proxy for study quality, which is assumed to be the only justified determinant of citation.

*Table 6. Univariate logistic regression analyses of possible determinants of selective citation*

| Determinant | **Cited article** | **Concordance** |
| --- | --- | --- |
| Significance BPA and health | X | X |
| In line with hypothesis negative health effect BPA | X | X |
| Study design | X | X |
| Sample size | X | X |
| Number of co-authors | X | X |
| Journal Impact Factor | X | X |
| Funding source | X | X |
| Continent | X | X |
| Affiliation corresponding author | X | X |
| Gender corresponding author | X | X |
| Study quality | X | X |
| Self-citation | - | X |
| Title of publication | X | X |

**Concordance**

In addition to testing the article characteristics of the cited article as determinants of citation, we want to see if article characteristics of the citing article are often in concordance with the characteristics of the cited article. When investigating the potential impact of the concordance between the characteristics of the cited and citing articles on being cited, we will perform a fixed effect logistic regression analysis.

**References**

1. Bornmann L, Daniel H-D. What do citation counts measure? A review of studies on citing behavior. Journal of Documentation. 2008;64(1):45-80.

2. Jannot A-S, Agoritsas T, Gayet-Ageron A, Perneger TV. Citation bias favoring statistically significant studies was present in medical research. Journal of Clinical Epidemiology. 2013 3//;66(3):296-301.

3. Bouter LM, Tijdink J, Axelsen N, Martinson BC, ter Riet G. Ranking major and minor research misbehaviors: results from a survey among participants of four World Conferences on Research Integrity. Research Integrity and Peer Review. 2016;1(1):17.

4. Greenberg SA. How citation distortions create unfounded authority: analysis of a citation network. Bmj. 2009;339:b2680.

5. Fanelli D. Positive results receive more citations, but only in some disciplines. Scientometrics. 2013;94(2):701-9.

6. Rubin BS. Bisphenol A: an endocrine disruptor with widespread exposure and multiple effects. The Journal of steroid biochemistry and molecular biology. 2011;127(1):27-34.

7. LaKind JS, Goodman M, Mattison DR. Bisphenol A and indicators of obesity, glucose metabolism/type 2 diabetes and cardiovascular disease: a systematic review of epidemiologic research. Critical reviews in toxicology. 2014;44(2):121-50.

8. Rochester JR. Bisphenol A and human health: a review of the literature. Reproductive toxicology. 2013;42:132-55.

9. Bergman Å, Heindel JJ, Jobling S, Kidd KA, Zoeller RT. Endocrine Disrupting Chemicals-2012. 2012.

10. Etter J-F, Stapleton J. Citations to trials of nicotine replacement therapy were biased toward positive results and high-impact-factor journals. Journal of clinical epidemiology. 2009 2009 Aug (Epub 2009 Jan;62(8). PubMed PMID: MEDLINE:19128941. English.

11. Holtcamp W. Obesogens: an environmental link to obesity. Environmental health perspectives. 2012;120(2):A63.

12. Conen D, Torres J, Ridker PM. Differential citation rates of major cardiovascular clinical trials according to source of funding: a survey from 2000 to 2005. Circulation. 2008 2008 Sep 23 (Epub 2008 Sep;118(13). PubMed PMID: MEDLINE:18779441. English.

13. Allik J. Bibliometric Analysis of the Journal of Cross-Cultural Psychology During the First Ten Years of the New Millennium. Journal of Cross-Cultural Psychology. 2013 May;44(4):657-67. PubMed PMID: WOS:000330301000009.

14. Walters GD. Predicting subsequent citations to articles published in twelve crime-psychology journals: Author impact versus journal impact. Scientometrics. 2006 Dec;69(3):499-510. PubMed PMID: WOS:000242672200002.

**Protocol deviations**

During the process of performing the citation analysis on bisphenol A literature, some changes were made with regard to the initial research plan described in this protocol.

1. During the data analysis phase, it appeared that two studies were outliers in the sense that they received a very high number of citations each. In order to test their impact on the determinants of citation found in the overall analysis, we performed a sensitivity analysis in which these two publications were excluded. This sensitivity analysis was therefore a posthoc analysis.
2. In the study protocol it was planned to create a visualisation of the citation network, in order to get a general overview of the BPA network. However, due to the relative high number of publications and citations it was not possible to create a meaningful network visualisation with the proposed visualisation programme CitNetExplorer.
3. In the study protocol it was proposed that the overall analysis would be adjusted for the study design, sample size and the presence of an a priori hypothesis. During the data extraction phase, it became clear that is not always clear if the publication defined an a priori hypothesis or not. Therefore, we decided not to include this as a determinant, to avoid misinterpretation of information. With regard to the adjusted logistic regression model, it appeared that the study design and sample size were highly correlated, which made it likely to overadjusted if both variables were added to the model. We chose to adjust only for study design, because we feel that this is more related to the quality of the study than sample size. Additionally not all publications, e.g. narrative and systematic reviews, did not always have a sample size that could be measured. Therefore it was found most accurate to use only the study design as proxy for study quality in the adjusted models.
