# Supplementary material for: Selective citation in scientific literature on the human health effects of bisphenol A
Source: Res Integr Peer Rev. 2019 Mar 29;4:6. doi: 10.1186/s41073-019-0065-7 (PMC6440006; doi:10.1186/s41073-019-0065-7)
Supplement: Supplementary file 2 — Table S1. Effect of concordance between the cited and citing article on the likelihood of being cited. Reference list bisphenol A network. (DOCX 80 kb) [file 41073_2019_65_MOESM2_ESM.docx]

**Selective citation in scientific literature on the human health effects of bisphenol A**

MJE Urlings ^1^*, B Duyx ^1^, G.M.H. Swaen ^1^, L.M. Bouter, ^2,3^, M.P. Zeegers,^1,4^

^1^ NUTRIM School of Nutrition and Translational Research in Metabolism, Maastricht University, Maastricht, The Netherlands

^2^ Department of Epidemiology and Biostatistics, VU University Medical Center, Amsterdam, The Netherlands

^3^ Department of Philosophy, Faculty of Humanities, Vrije Universiteit, Amsterdam, The Netherlands

^4^ CAPHRI School for Public Health and Primary Care, Maastricht University, Maastricht, The Netherlands

* Corresponding author: mje.urlings@maastrichtuniversity.nl

- Digital supplement -

**Content**

Table S1. Effect of concordance between the cited and citing article on the likelihood of being cited

Reference list bisphenol A network

*Table S1: Effect of concordance between the cited and citing article on the likelihood of being cited*

| **Variable** | **Crude OR** | **Adjusted OR*** |
| --- | --- | --- |
| Statistical significance | 0.78 (0.58-1.04) | 0.70 (0.49-1.02) |
| In line with hypothesis | 0.89 (0.70-1.13) | 1.06 (0.79-1.42) |
| Health outcomes | 3.48 (3.01-4.03) | 3.97 (3.40-4.70) |
| Study design | 1.42 (1.22-1.66) | - |
| Sample size** | 1.59 (1.29-1.96) | 1.59 (1.29-1.96) |
| Journal impact factor** | 1.21 (1.01-1.44) | 1.30 (1.07-1.58) |
| Funding source | 1.70 (1.39-2.09) | 1.53 (1.21-1.93) |
| Number of references** | 0.87 (0.75-1.02) | 0.85 (0.72-1.01) |
| Title of publication | 1.15 (0.98-1.34) | 1.03 (0.87-1.22) |
| Gender | 1.04 (0.90-1.21) | 1.10 (0.94-1.30) |
| Affiliation corresponding author | 0.93 (0.78-1.11) | 0.88 (0.72-1.07) |

** Adjusted for study design; ** for this analysis the continuous variables are converted into three categories based on tertiles*

**Reference list bisphenol A network**

1. Takeuchi T, Tsutsumi O. Serum bisphenol A concentrations showed gender differences, possibly linked to androgen levels. Biochemical and Biophysical Research Communications. 2002 Feb;291(1):76-8.

2. Takeuchi T, Tsutsumi O, Ikezuki Y, Takai Y, Taketani Y. Positive relationship between androgen and the endocrine disruptor, bisphenol A, in normal women and women with ovarian dysfunction. Endocrine Journal. 2004 Apr;51(2):165-9.

3. Tsutsumi O. Assessment of human contamination of estrogenic endocrine- disrupting chemicals and their risk for human reproduction. Journal of Steroid Biochemistry and Molecular Biology. 2005 Feb;93(2-5):325-30.

4. Dash C, Marcus M, Terry PD. Bisphenol A: Do recent studies of health effects among humans inform the long-standing debate? Mutation Research- Reviews in Mutation Research. 2006 Nov-Dec;613(2-3):68-75.

5. Yang M, Kim SY, Chang SS, Lee IS, Kawamoto T. Urinary concentrations of bisphenol a in relation to biomarkers of sensitivity and effect and endocrine- related health effects. Environmental and Molecular Mutagenesis. 2006 Oct; 47(8):571-8.

6. Vandenberg LN, Hauser R, Marcus M, Olea N, Welshons WV. Human exposure to bisphenol A (BPA). Reproductive Toxicology. 2007 Aug-Sep;24(2): 139-77.

7. Lang IA, Galloway TS, Scarlett A, Henley WE, Depledge M, Wallace RB, et al. Association of urinary bisphenol A concentration with medical disorders and laboratory abnormalities in adults. Jama-Journal of the American Medical Association. 2008 Sep;300(11):1303-10.

8. Padmanabhan V, Siefert K, Ransom S, Johnson T, Pinkerton J, Anderson L, et al. Maternal bisphenol-A levels at delivery: a looming problem? Journal of Perinatology. 2008 Apr;28(4):258-63.

9. Hong YC, Park EY, Park MS, Ko JA, Oh SY, Kim H, et al. Community level exposure to chemicals and oxidative stress in adult population. Toxicology Letters. 2009 Jan;184(2):139-44.

10. Meeker JD, Sathyanarayana S, Swan SH. Phthalates and other additives in plastics: human exposure and associated health outcomes. Philosophical Transactions of the Royal Society B-Biological Sciences. 2009 Jul;364(1526): 2097-113.

11. Yang M, Ryu JH, Jeon R, Kang D, Yoo KY. Effects of bisphenol A on breast cancer and its risk factors. Archives of Toxicology. 2009 Mar;83(3):281-5.

12. Cantonwine D, Meeker JD, Hu H, Sanchez BN, Lamadrid-Figueroa H, Mercado-Garcia A, et al. Bisphenol a exposure in Mexico City and risk of prematurity: a pilot nested case control study. Environmental Health. 2010 Oct;9.

13. Galloway T, Cipelli R, Guralnik J, Ferrucci L, Bandinelli S, Corsi AM, et al. Daily Bisphenol A Excretion and Associations with Sex Hormone Concentrations: Results from the InCHIANTI Adult Population Study. Environmental Health Perspectives. 2010 Nov;118(11):1603-8.

14. Li D, Zhou Z, Qing D, He Y, Wu T, Miao M, et al. Occupational exposure to bisphenol-A (BPA) and the risk of Self-Reported Male Sexual Dysfunction. Human Reproduction. 2010 Feb;25(2):519-27.

15. Li DK, Zhou ZJ, Miao MH, He YH, Qing DD, Wu TJ, et al. Relationship Between Urine Bisphenol-A Level and Declining Male Sexual Function. Journal of Andrology. 2010 Sep-Oct;31(5):500-6.

16. Meeker JD. Exposure to environmental endocrine disrupting compounds and men's health. Maturitas. 2010 Jul;66(3):236-41.

17. Meeker JD, Calafat AM, Hauser R. Urinary Bisphenol A Concentrations in Relation to Serum Thyroid and Reproductive Hormone Levels in Men from an Infertility Clinic. Environmental Science & Technology. 2010 Feb;44(4):1458-63.

18. Meeker JD, Ehrlich S, Toth TL, Wright DL, Calafat AM, Trisini AT, et al. Semen quality and sperm DNA damage in relation to urinary bisphenol A among men from an infertility clinic. Reproductive Toxicology. 2010 Dec;30(4):532-9.

19. Melzer D, Rice NE, Lewis C, Henley WE, Galloway TS. Association of Urinary Bisphenol A Concentration with Heart Disease: Evidence from NHANES 2003/06. Plos One. 2010 Jan;5(1).

20. Mendiola J, Jorgensen N, Andersson AM, Calafat AM, Ye XY, Redmon JB, et al. Are Environmental Levels of Bisphenol A Associated with Reproductive Function in Fertile Men? Environmental Health Perspectives. 2010 Sep;118(9): 1286-91.

21. Mok-Lin E, Ehrlich S, Williams PL, Petrozza J, Wright DL, Calafat AM, et al. Urinary bisphenol A concentrations and ovarian response among women undergoing IVF. International Journal of Andrology. 2010 Apr;33(2):385-93.

22. Balabanic D, Rupnik M, Klemencic AK. Negative impact of endocrine- disrupting compounds on human reproductive health. Reproduction Fertility and Development. 2011;23(3):403-16.

23. Bloom MS, Saal FSV, Kim D, Taylor JA, Lamb JD, Fujimoto VY. Serum unconjugated bisphenol A concentrations in men may influence embryo quality indicators during in vitro fertilization. Environmental Toxicology and Pharmacology. 2011 Sep;32(2):319-23.

24. Braun JM, Hauser R. Bisphenol A and children's health. Current Opinion in Pediatrics. 2011 Apr;23(2):233-9.

25. Carwile JL, Michels KB. Urinary bisphenol A and obesity: NHANES 2003-2006. Environmental Research. 2011 Aug;111(6):825-30.

26. Caserta D, Mantovani A, Marci R, Fazi A, Ciardo F, La Rocca C, et al. Environment and women's reproductive health. Human Reproduction Update. 2011 May-Jun;17(3):418-33.

27. Clayton EMR, Todd M, Dowd JB, Aiello AE. The Impact of Bisphenol A and Triclosan on Immune Parameters in the U.S. Population, NHANES 2003-2006. Environmental Health Perspectives. 2011 Mar;119(3):390-6.

28. Meeker JD, Ferguson KK. Relationship between Urinary Phthalate and Bisphenol A Concentrations and Serum Thyroid Measures in US Adults and Adolescents from the National Health and Nutrition Examination Survey (NHANES) 2007-2008. Environmental Health Perspectives. 2011 Oct;119(10): 1396-402.

29. Meeker JD, Yang T, Ye XY, Calafat AM, Hauser R. Urinary Concentrations of Parabens and Serum Hormone Levels, Semen Quality Parameters, and Sperm DNA Damage. Environmental Health Perspectives. 2011 Feb;119(2):252-7.

30. Melzer D, Galloway T. Bisphenol A and Adult Disease: Making Sense of Fragmentary Data and Competing Inferences. Annals of Internal Medicine. 2011 Sep;155(6):392-U86. PubMed PMID: WOS:000295033400020. 31. Miao MH, Yuan W, He YH, Zhou ZJ, Wang JT, Gao ES, et al. In Utero Exposure to Bisphenol-A and Anogenital Distance of Male Offspring. Birth Defects Research Part a-Clinical and Molecular Teratology. 2011 Oct;91(10): 867-72.

32. Shankar A, Teppala S. Relationship between Urinary Bisphenol A Levels and Diabetes Mellitus. Journal of Clinical Endocrinology & Metabolism. 2011 Dec;96(12):3822-6. PubMed PMID: WOS:000298295200057. 33. Silver MK, O'Neill MS, Sowers MR, Park SK. Urinary Bisphenol A and Type-2 Diabetes in US Adults: Data from NHANES 2003-2008. Plos One. 2011 Oct;6(10).

34. You L, Zhu XZ, Shrubsole MJ, Fan H, Chen J, Dong J, et al. Renal Function, Bisphenol A, and Alkylphenols: Results from the National Health and Nutrition Examination Survey (NHANES 2003-2006). Environmental Health Perspectives. 2011 Apr;119(4):527-33.

35. de Cock M, Maas YGH, van de Bor M. Does perinatal exposure to endocrine disruptors induce autism spectrum and attention deficit hyperactivity disorders? Review. Acta Paediatrica. 2012 Aug;101(8):811-8.

36. Ehrlich S, Williams PL, Missmer SA, Flaws JA, Berry KF, Calafat AM, et al. Urinary Bisphenol A Concentrations and Implantation Failure among Women Undergoing in Vitro Fertilization. Environmental Health Perspectives. 2012 Jul; 120(7):978-83.

37. Ehrlich S, Williams PL, Missmer SA, Flaws JA, Ye XY, Calafat AM, et al. Urinary bisphenol A concentrations and early reproductive health outcomes among women undergoing IVF. Human Reproduction. 2012 Dec;27(12):3583-92.

38. Fenichel P, Dechaux H, Harthe C, Gal J, Ferrari P, Pacini P, et al. Unconjugated bisphenol A cord blood levels in boys with descended or undescended testes. Human Reproduction. 2012 Apr;27(4):983-90.

39. Hanna CW, Bloom MS, Robinson WP, Kim D, Parsons PJ, Saal FSV, et al. DNA methylation changes in whole blood is associated with exposure to the environmental contaminants, mercury, lead, cadmium and bisphenol A, in women undergoing ovarian stimulation for IVF. Human Reproduction. 2012 May;27(5): 1401-10.

40. Li MA, Bi YF, Qi L, Wang TG, Xu M, Huang Y, et al. Exposure to bisphenol A is associated with low-grade albuminuria in Chinese adults. Kidney International. 2012 Jun;81(11):1131-9.

41. Lind L, Lind PM. Can persistent organic pollutants and plastic-associated chemicals cause cardiovascular disease? Journal of Internal Medicine. 2012 Jun; 271(6):537-53.

42. Olsen L, Lind L, Lind PM. Associations between circulating levels of bisphenol A and phthalate metabolites and coronary risk in the elderly. Ecotoxicology and Environmental Safety. 2012 Jun;80:179-83.

43. Philippat C, Mortamais M, Chevrier C, Petit C, Calafat AM, Ye XY, et al. Exposure to Phthalates and Phenols during Pregnancy and Offspring Size at Birth. Environmental Health Perspectives. 2012 Mar;120(3):464-70.

44. Polanska K, Jurewicz J, Hanke W. Exposure to environmental and lifestyle factors and attention-deficit / hyperactivity disorder in children - A review of epidemiological studies. International Journal of Occupational Medicine and Environmental Health. 2012;25(4):330-55.

45. Shankar A, Teppala S, Sabanayagam C. Bisphenol A and Peripheral Arterial Disease: Results from the NHANES. Environmental Health Perspectives. 2012 Sep;120(9):1297-300.

46. Spanier AJ, Kahn RS, Kunselman AR, Hornung R, Xu YY, Calafat AM, et al. Prenatal Exposure to Bisphenol A and Child Wheeze from Birth to 3 Years of Age. Environmental Health Perspectives. 2012 Jun;120(6):916-20.

47. Wang TG, Li MA, Chen B, Xu M, Xu Y, Huang Y, et al. Urinary Bisphenol A (BPA) Concentration Associates with Obesity and Insulin Resistance. Journal of Clinical Endocrinology & Metabolism. 2012 Feb;97(2):E223-E7.

48. Zhao HY, Bi YF, Ma LY, Zhao L, Wang TG, Zhang LZ, et al. The effects of bisphenol A (BPA) exposure on fat mass and serum leptin concentrations have no impact on bone mineral densities in non-obese premenopausal women. Clinical Biochemistry. 2012 Dec;45(18):1602-6.

49. Sharpe RM, Drake AJ. Obesogens and Obesity-an Alternative View? Obesity. 2013 Jun;21(6):1081-3.

50. Casey MF, Neidell M. Disconcordance in Statistical Models of Bisphenol A and Chronic Disease Outcomes in NHANES 2003-08. Plos One. 2013 Nov;8(11).

51. Chen MJ, Tang R, Fu GB, Xu B, Zhu PF, Qiao SL, et al. Association of exposure to phenols and idiopathic male infertility. Journal of Hazardous Materials. 2013 Apr;250:115-21.

52. Chen XJ, Chen MJ, Xu B, Tang R, Han XM, Qin YF, et al. Parental phenols exposure and spontaneous abortion in Chinese population residing in the middle and lower reaches of the Yangtze River. Chemosphere. 2013 Sep;93(2):217-22.

53. Chevrier J, Gunier RB, Bradman A, Holland NT, Calafat AM, Eskenazi B, et al. Maternal Urinary Bisphenol A during Pregnancy and Maternal and Neonatal Thyroid Function in the CHAMACOS Study. Environmental Health Perspectives. 2013 Jan;121(1):138-44.

54. Ehrlich S, Williams PL, Hauser R, Missmer SA, Peretz J, Calafat AM, et al. Urinary bisphenol A concentrations and cytochrome P450 19 A1 (Cyp19) gene expression in ovarian granulosa cells: An in vivo human study. Reproductive Toxicology. 2013 Dec;42:18-23.

55. Eng DS, Lee JM, Gebremariam A, Meeker JD, Peterson K, Padmanabhan V. Bisphenol A and Chronic Disease Risk Factors in US Children. Pediatrics. 2013 Sep;132(3):E637-E45.

56. Harley KG, Gunier RB, Kogut K, Johnson C, Bradman A, Calafat AM, et al. Prenatal and early childhood bisphenol A concentrations and behavior in school- aged children. Environmental Research. 2013 Oct;126:43-50.

57. Hong SB, Hong YC, Kim JW, Park EJ, Shin MS, Kim BN, et al. Bisphenol A in relation to behavior and learning of school-age children. Journal of Child Psychology and Psychiatry. 2013 Aug;54(8):890-9.

58. Kim JH, Rozek LS, Soliman AS, Sartor MA, Hablas A, Seifeldin IA, et al. Bisphenol A-associated epigenomic changes in prepubescent girls: a cross- sectional study in Gharbiah, Egypt. Environmental Health. 2013 Apr;12.

59. Kim K, Park H. Association between urinary concentrations of bisphenol A and type 2 diabetes in Korean adults: A population-based cross-sectional study. International Journal of Hygiene and Environmental Health. 2013 Jul;216(4): 467-71.

60. Li DK, Miao MH, Zhou ZH, Wu CH, Shi HJ, Liu XQ, et al. Urine Bisphenol-A Level in Relation to Obesity and Overweight in School-Age Children. Plos One. 2013 Jun;8(6).

61. Louis GMB, Peterson CM, Chen Z, Croughan M, Sundaram R, Stanford J, et al. Bisphenol A and phthalates and endometriosis: the Endometriosis: Natural History, Diagnosis and Outcomes Study. Fertility and Sterility. 2013 Jul;100(1): 162-+.

62. Rochester JR. Bisphenol A and human health: A review of the literature. Reproductive Toxicology. 2013 Dec;42:132-55.

63. Shen Y, Xu Q, Ren ML, Feng X, Cai YL, Gao YX. Measurement of Phenolic Environmental Estrogens in Women with Uterine Leiomyoma. Plos One. 2013 Nov;8(11).

64. Sprague BL, Trentham-Dietz A, Hedman CJ, Wang J, Hemming JDC, Hampton JM, et al. Circulating serum xenoestrogens and mammographic breast density. Breast Cancer Research. 2013;15(3).

65. Sriphrapradang C, Chailurkit LO, Aekplakorn W, Ongphiphadhanakul B. Association between bisphenol A and abnormal free thyroxine level in men. Endocrine. 2013 Oct;44(2):441-7.

66. Valvi D, Casas M, Mendez MA, Ballesteros-Gomez A, Luque N, Rubio S, et al. Prenatal Bisphenol A Urine Concentrations and Early Rapid Growth and Overweight Risk in the Offspring. Epidemiology. 2013 Nov;24(6):791-9.

67. Volberg V, Harley K, Calafat AM, Dave V, McFadden J, Eskenazi B, et al. Maternal Bisphenol A Exposure During Pregnancy and Its Association With Adipokines in Mexican-American Children. Environmental and Molecular Mutagenesis. 2013 Oct;54(8):621-8.

68. Yum T, Lee S, Kim Y. Association between precocious puberty and some endocrine disruptors in human plasma. Journal of Environmental Science and Health Part a-Toxic/Hazardous Substances & Environmental Engineering. 2013;48(8):912-7.

69. Zhou FQ, Zhang L, Liu A, Shen Y, Yuan JP, Yu XJ, et al. Measurement of phenolic environmental estrogens in human urine samples by HPLC-MS/MS and primary discussion the possible linkage with uterine leiomyoma. Journal of Chromatography B-Analytical Technologies in the Biomedical and Life Sciences. 2013 Nov;938:80-5.

70. Zhou Q, Miao MH, Ran MM, Ding L, Bai L, Wu TT, et al. Serum bisphenol-A concentration and sex hormone levels in men. Fertility and Sterility. 2013 Aug; 100(2):478-82.

71. Ahmadkhaniha R, Mansouri M, Yunesian M, Omidfar K, Jeddi MZ, Larijani B, et al. Association of urinary bisphenol a concentration with type-2 diabetes mellitus. Journal of Environmental Health Science and Engineering. 2014 Mar; 12.

72. Barrett ES, Sobolewski M. Polycystic Ovary Syndrome: Do Endocrine- Disrupting Chemicals Play a Role? Seminars in Reproductive Medicine. 2014 May;32(3):166-76.

73. Durmaz E, Asci A, Erkekoglu P, Akcurin S, Gumusel BK, Bircan I. Urinary Bisphenol A Levels in Girls with Idiopathic Central Precocious Puberty. Journal of Clinical Research in Pediatric Endocrinology. 2014;6(1):16-21.

74. Evans SF, Kobrosly RW, Barrett ES, Thurston SW, Calafat AM, Weiss B, et al. Prenatal bisphenol A exposure and maternally reported behavior in boys and girls. Neurotoxicology. 2014 Dec;45:91-9.

75. Gao XQ, Wang HS. Impact of Bisphenol A on the Cardiovascular System - Epidemiological and Experimental Evidence and Molecular Mechanisms. International Journal of Environmental Research and Public Health. 2014 Aug; 11(8):8399-413.

76. Khalil N, Chen AM, Lee M. Endocrine disruptive compounds and cardio- metabolic risk factors in children. Current Opinion in Pharmacology. 2014 Dec; 19:120-4.

77. Khalil N, Ebert JR, Wang L, Belcher S, Lee M, Czerwinski SA, et al. Bisphenol A and cardiometabolic risk factors in obese children. Science of the Total Environment. 2014 Feb;470:726-32.

78. Kim EJ, Lee D, Chung BC, Pyo H, Lee J. Association between urinary levels of bisphenol-A and estrogen metabolism in Korean adults. Science of the Total Environment. 2014 Feb;470:1401-7.

79. La Rocca C, Tait S, Guerranti C, Busani L, Ciardo F, Bergamasco B, et al. Exposure to Endocrine Disrupters and Nuclear Receptor Gene Expression in Infertile and Fertile Women from Different Italian Areas. International Journal of Environmental Research and Public Health. 2014 Oct;11(10):10146-64.

80. LaRocca J, Binder AM, McElrath TF, Michels KB. The impact of first trimester phthalate and phenol exposure on IGF2/H19 genomic imprinting and birth outcomes. Environmental Research. 2014 Aug;133:396-406.

81. Lassen TH, Frederiksen H, Jensen TK, Petersen JH, Joensen UN, Main KM, et al. Urinary Bisphenol A Levels in Young Men: Association with Reproductive Hormones and Semen Quality. Environmental Health Perspectives. 2014 May;122(5):478-84.

82. Lee BE, Park H, Hong YC, Ha M, Kim Y, Chang N, et al. Prenatal bisphenol A and birth outcomes: MOCEH (Mothers and Children's Environmental Health) study. International Journal of Hygiene and Environmental Health. 2014 Mar; 217(2-3):328-34.

83. Lee MR, Park H, Bae S, Lim YH, Kim JH, Cho SH, et al. Urinary bisphenol A concentrations are associated with abnormal liver function in the elderly: a repeated panel study. Journal of Epidemiology and Community Health. 2014 Apr; 68(4):312-7.

84. Louis GMB, Sundaram R, Sweeney AM, Schisterman EF, Maisog J, Kannan K. Urinary bisphenol A, phthalates, and couple fecundity: the Longitudinal Investigation of Fertility and the Environment (LIFE) Study. Fertility and Sterility. 2014 May;101(5):1359-66.

85. Machtinger R, Orvieto R. Bisphenol A, oocyte maturation, implantation, and IVF outcome: review of animal and human data. Reproductive Biomedicine Online. 2014 Oct;29(4):404-10.

86. Miao M, Zhou X, Li Y, Zhang O, Zhou Z, Li T, et al. LINE-1 hypomethylation in spermatozoa is associated with Bisphenol A exposure. Andrology. 2014 Jan; 2(1):138-44.

87. Michalowicz J. Bisphenol A - Sources, toxicity and biotransformation. Environmental Toxicology and Pharmacology. 2014 Mar;37(2):738-58.

88. Mileva G, Baker SL, Konkle ATM, Bielajew C. Bisphenol-A: Epigenetic Reprogramming and Effects on Reproduction and Behavior. International Journal of Environmental Research and Public Health. 2014 Jul;11(7):7537-61.

89. Mirmira P, Evans-Molina C. Bisphenol A, obesity, and type 2 diabetes mellitus: genuine concern or unnecessary preoccupation? Translational Research. 2014 Jul;164(1):13-21.

90. Peretz J, Vrooman L, Ricke WA, Hunt PA, Ehrlich S, Hauser R, et al. Bisphenol A and Reproductive Health: Update of Experimental and Human Evidence, 2007-2013. Environmental Health Perspectives. 2014 Aug;122(8): 775-86.

91. Philippat C, Botton J, Calafat AM, Ye XY, Charles MA, Slama R, et al. Prenatal Exposure to Phenols and Growth in Boys. Epidemiology. 2014 Sep; 25(5):625-35.

92. Posnack NG. The Adverse Cardiac Effects of Di(2-ethylhexyl)phthalate and Bisphenol A. Cardiovascular Toxicology. 2014 Dec;14(4):339-57.

93. Rezg R, El-Fazaa S, Gharbi N, Mornagui B. Bisphenol A and human chronic diseases: Current evidences, possible mechanisms, and future perspectives. Environment International. 2014 Mar;64:83-90.

94. Ronn M, Lind L, Orberg J, Kullberg J, Soderberg S, Larsson A, et al. Bisphenol A is related to circulating levels of adiponectin, leptin and ghrelin, but not to fat mass or fat distribution in humans. Chemosphere. 2014 Oct;112:42-8.

95. Shiue I. Higher urinary heavy metal, arsenic, and phthalate concentrations in people with high blood pressure: US NHANES, 2009-2010. Blood Pressure. 2014 Dec;23(6):363-9.

96. Song Y, Hauser R, Hu FB, Franke AA, Liu S, Sun Q. Urinary concentrations of bisphenol A and phthalate metabolites and weight change: a prospective investigation in US women. International Journal of Obesity. 2014 Dec;38(12): 1532-7.

97. Tarapore P, Ying J, Ouyang B, Burke B, Bracken B, Ho SM. Exposure to Bisphenol A Correlates with Early-Onset Prostate Cancer and Promotes Centrosome Amplification and Anchorage-Independent Growth In Vitro. Plos One. 2014 Mar;9(3).

98. Trabert B, Falk R, Figueroa J, Graubard B, Garcia-Closas M, Lissowska J, et al. ' Urinary bisphenol A-glucuronide and postmenopausal breast cancer in Poland. Cancer Causes & Control. 2014 Dec;25(12):1587-93.

99. Troisi J, Mikelson C, Richards S, Symes S, Adair D, Zullo F, et al. Placental concentrations of bisphenol A and birth weight from births in the Southeastern US. Placenta. 2014 Nov;35(11):947-52.

100. Upson K, Sathyanarayana S, De Roos AJ, Koch HM, Scholes D, Holt VL. A population-based case-control study of urinary bisphenol A concentrations and risk of endometriosis. Human Reproduction. 2014 Nov;29(11):2457-64.

101. Vagi SJ, Azziz-Baumgartner E, Sjodin A, Calafat AM, Dumesic D, Gonzalez L, et al. Exploring the potential association between brominated diphenyl ethers, polychlorinated biphenyls, organochlorine pesticides, perfluorinated compounds, phthalates, and bisphenol a in polycystic ovary syndrome: a case-control study. Bmc Endocrine Disorders. 2014 Oct;14.

102. Aekplakorn W, Chailurkit LO, Ongphiphadhanakul B. Relationship of serum bisphenol A with diabetes in the Thai population, National Health Examination Survey IV, 2009. Journal of Diabetes. 2015 Mar;7(2):240-9.

103. Andra SS, Kalyvas H, Andrianou XD, Charisiadis P, Christophi CA, Makris KC. Preliminary evidence of the association between monochlorinated bisphenol A exposure and type II diabetes mellitus: A pilot study. Journal of Environmental Science and Health Part a-Toxic/Hazardous Substances & Environmental Engineering. 2015;50(3):243-59.

104. Andra SS, Makris KC. Association between urinary levels of bisphenol A and its monochlorinated derivative and obesity. Journal of Environmental Science and Health Part a-Toxic/Hazardous Substances & Environmental Engineering. 2015 Sep;50(11):1169-79.

105. Bae J, Kim S, Kannan K, Louis GMB. Couples' urinary bisphenol A and phthalate metabolite concentrations and the secondary sex ratio. Environmental Research. 2015 Feb;137:450-7.

106. Berghuis SA, Bos AF, Sauer PJJ, Roze E. Developmental neurotoxicity of persistent organic pollutants: an update on childhood outcome. Archives of Toxicology. 2015 May;89(5):687-709.

107. Bertoli S, Leone A, Battezzati A. Human Bisphenol A Exposure and the "Diabesity Phenotype". Dose-Response. 2015 Jul-Sep;13(3).

108. Chevalier N, Brucker-Davis F, Lahlou N, Coquillard P, Pugeats M, Pacini P, et al. A negative correlation between insulin-like peptide 3 and bisphenol A in human cord blood suggests an effect of endocrine disruptors on testicular descent during fetal development. Human Reproduction. 2015 Feb;30(2):447-53.

109. Chevalier N, Fenichel P. Bisphenol A: Targeting metabolic tissues. Reviews in Endocrine & Metabolic Disorders. 2015 Dec;16(4):299-309.

110. De Felice B, Manfellotto F, Palumbo A, Troisi J, Zullo F, Di Carlo C, et al. Genome-wide microRNA expression profiling in placentas from pregnant women exposed to BPA. Bmc Medical Genomics. 2015 Sep;8.

111. Den Hond E, Tournaye H, De Sutter P, Ombelet W, Baeyens W, Covaci A, et al. Human exposure to endocrine, disrupting chemicals and fertility: A case- control study in male subfertility patients. Environment International. 2015 Nov; 84:154-60.

112. Dodge LE, Williams PL, Williams MA, Missmer SA, Toth TL, Calafat AM, et al. Paternal Urinary Concentrations of Parabens and Other Phenols in Relation to Reproductive Outcomes among Couples from a Fertility Clinic. Environmental Health Perspectives. 2015 Jul;123(7):665-71.

113. Goldstone AE, Chen Z, Perry MJ, Kannan K, Louis GMB. Urinary bisphenol A and semen quality, the LIFE Study. Reproductive Toxicology. 2015 Jan; 51:7-13.

114. Guida M, Troisi J, Ciccone C, Granozio G, Cosimato C, Sardo AD, et al. Bisphenol A and congenital developmental defects in humans. Mutation Research-Fundamental and Molecular Mechanisms of Mutagenesis. 2015 Apr; 774:33-9.

115. Huo WQ, Xia W, Wan YJ, Zhang B, Zhou AF, Zhang YM, et al. Maternal urinary bisphenol A levels and infant low birth weight: A nested case-control study of the Health Baby Cohort in China. Environment International. 2015 Dec; 85:96-103. PubMed PMID: WOS:000365363800011.

116. Inadera H. Neurological Effects of Bisphenol A and its Analogues. International Journal of Medical Sciences. 2015;12(12):926-36.

117. Le Corre L, Besnard P, Chagnon MC. BPA, an Energy Balance Disruptor. Critical Reviews in Food Science and Nutrition. 2015;55(6):769-77.

118. Li Q, Kappil MA, Li A, Dassanayake PS, Darrah TH, Friedman AE, et al. Exploring the associations between microRNA expression profiles and environmental pollutants in human placenta from the National Children's Study (NCS). Epigenetics. 2015 Sep;10(9):793-802.

119. Lin CY, Shen FY, Lian GW, Chien KL, Sung FC, Chen PC, et al. Association between levels of serum bisphenol A, a potentially harmful chemical in plastic containers, and carotid artery intima-media thickness in adolescents and young adults. Atherosclerosis. 2015 Aug;241(2):657-63.

120. Liu XQ, Miao MH, Zhou ZJ, Gao ES, Chen JP, Wang JT, et al. Exposure to bisphenol-A and reproductive hormones among male adults. Environmental Toxicology and Pharmacology. 2015 Mar;39(2):934-41.

121. Minguez-Alarcon L, Gaskins AJ, Chiu YH, Williams PL, Ehrlich S, Chavarro JE, et al. Urinary bisphenol A concentrations and association with in vitro fertilization outcomes among women from a fertility clinic. Human Reproduction. 2015 Sep;30(9):2120-8.

122. Mustieles V, Perez-Lobato R, Olea N, Fernandez MF. Bisphenol A: Human exposure and neurobehavior. Neurotoxicology. 2015 Jul;49:174-84.

123. Oppeneer SJ, Robien K. Bisphenol A exposure and associations with obesity among adults: a critical review. Public Health Nutrition. 2015 Jul;18(10): 1847-63.

124. Palioura E, Diamanti-Kandarakis E. Polycystic ovary syndrome (PCOS) and endocrine disrupting chemicals (EDCs). Reviews in Endocrine & Metabolic Disorders. 2015 Dec;16(4):365-71.

125. Pollack AZ, Louis GMB, Chen Z, Sun L, Trabert B, Guo Y, et al. Bisphenol A, benzophenone-type ultraviolet filters, and phthalates in relation to uterine leiomyoma. Environmental Research. 2015 Feb;137:101-7.

126. Pornkunwilai S, Nosoongnoen W, Jantarat C, Wachrasindhu S, Supornsilchai V. Urinary bisphenol A detection is significantly associated with young and obese Thai children. Asian Biomedicine. 2015 Jun;9(3):363-72.

127. Ranciere F, Lyons JG, Loh VHY, Botton J, Galloway T, Wang TG, et al. Bisphenol A and the risk of cardiometabolic disorders: a systematic review with meta-analysis of the epidemiological evidence. Environmental Health. 2015 May; 14.

128. Savastano S, Tarantino G, D'Esposito V, Passaretti F, Cabaro S, Liotti A, et al. Bisphenol-A plasma levels are related to inflammatory markers, visceral obesity and insulin-resistance: a cross-sectional study on adult male population. Journal of Translational Medicine. 2015 May;13.

129. Shen YP, Zheng YM, Jiang JT, Liu YM, Luo XM, Shen Z, et al. Higher Urinary Bisphenol A Concentration Is Associated with Unexplained Recurrent Miscarriage Risk: Evidence from a Case-Control Study in Eastern China. Plos One. 2015 May;10(5).

130. Watkins DJ, Ferguson KK, Del Toro LVA, Alshawabkeh AN, Cordero JF, Meeker JD. Associations between urinary phenol and paraben concentrations and markers of oxidative stress and inflammation among pregnant women in Puerto Rico. International Journal of Hygiene and Environmental Health. 2015 Mar;218(2):212-9.

131. Xue JC, Wu Q, Sakthivel S, Pavithran PV, Vasukutty JR, Kannan K. Urinary levels of endocrine-disrupting chemicals, including bisphenols, bisphenol A diglycidyl ethers, benzophenones, parabens, and triclosan in obese and non- obese Indian children. Environmental Research. 2015 Feb;137:120-8.

132. Yuan M, Bai MZ, Huang XF, Zhang Y, Liu J, Hu MH, et al. Preimplantation Exposure to Bisphenol A and Triclosan May Lead to Implantation Failure in Humans. Biomed Research International. 2015.

133. Zhuang WL, Wu KS, Wang YK, Zhu HJ, Deng ZZ, Peng L, et al. Association of Serum Bisphenol-A Concentration and Male Reproductive Function Among Exposed Workers. Archives of Environmental Contamination and Toxicology. 2015 Jan;68(1):38-45.

134. Aker AM, Watkins DJ, Johns LE, Ferguson KK, Soldin OP, Del Toro LVA, et al. Phenols and parabens in relation to reproductive and thyroid hormones in pregnant women. Environmental Research. 2016 Nov;151:30-7.

135. Andrianou XD, Gangler S, Piciu A, Charisiadis P, Zira C, Aristidou K, et al. Human Exposures to Bisphenol A, Bisphenol F and Chlorinated Bisphenol A Derivatives and Thyroid Function. Plos One. 2016 Oct;11(10).

136. Bulus AD, Asci A, Erkekoglu P, Balci A, Andiran N, Kocer-Gumusel B. The evaluation of possible role of endocrine disruptors in central and peripheral precocious puberty. Toxicology Mechanisms and Methods. 2016 Sep;26(7): 493-500.

137. Chailurkit LO, Aekplakorn W, Ongphiphadhanakul B. The Association of Serum Bisphenol A with Thyroid Autoimmunity. International Journal of Environmental Research and Public Health. 2016 Nov;13(11).

138. Ferguson KK, Cantonwine DE, McElrath TF, Mukherjee B, Meeker JD. Repeated measures analysis of associations between urinary bisphenol-A concentrations and biomarkers of inflammation and oxidative stress in pregnancy. Reproductive Toxicology. 2016 Dec;66:93-8.

139. Fernandez MF, Arrebola JP, Jimenez-Diaz I, Saenz JM, Molina-Molina JM, Ballesteros O, et al. Bisphenol A and other phenols in human placenta from children with cryptorchidism or hypospadias. Reproductive Toxicology. 2016 Jan; 59:89-95.

140. Giesbrecht GF, Liu JY, Ejaredar M, Dewey D, Letourneau N, Campbell T, et al. Urinary bisphenol A is associated with dysregulation of HPA-axis function in pregnant women: Findings from the APrON cohort study. Environmental Research. 2016 Nov;151:689-97.

141. Giulivo M, de Alda ML, Capri E, Barcelo D. Human exposure to endocrine disrupting compounds: Their role in reproductive systems, metabolic syndrome and breast cancer. A review. Environmental Research. 2016 Nov;151:251-64.

142. Han C, Hong YC. Bisphenol A, Hypertension, and Cardiovascular Diseases: Epidemiological, Laboratory, and Clinical Trial Evidence. Current Hypertension Reports. 2016 Jan;18(2).

143. Hoepner LA, Whyatt RM, Widen EM, Hassoun A, Oberfield SE, Mueller NT, et al. Bisphenol A and Adiposity in an Inner-City Birth Cohort. Environmental Health Perspectives. 2016 Oct;124(10):1644-50.

144. Jagne J, White D, Jefferson F. Endocrine-Disrupting Chemicals: Adverse Effects of Bisphenol A and Parabens to Women's Health. Water Air and Soil Pollution. 2016 Jun;227(6).

145. Johns LE, Ferguson KK, Meeker JD. Relationships Between Urinary Phthalate Metabolite and Bisphenol A Concentrations and Vitamin D Levels in US Adults: National Health and Nutrition Examination Survey (NHANES), 2005-2010. Journal of Clinical Endocrinology & Metabolism. 2016 Nov;101(11): 4062-9.

146. Jukic AM, Calafat AM, McConnaughey DR, Longnecker MP, Hoppin JA, Weinberg CR, et al. Urinary Concentrations of Phthalate Metabolites and Bisphenol A and Associations with Follicular-Phase Length, Luteal-Phase Length, Fecundability, and Early Pregnancy Loss. Environmental Health Perspectives. 2016 Mar;124(3):321-8.

147. LaRocca J, Binder AM, McElrath TF, Michels KB. First-Trimester Urine Concentrations of Phthalate Metabolites and Phenols and Placenta miRNA Expression in a Cohort of US Women. Environmental Health Perspectives. 2016 Mar;124(3):380-7.

148. Liao SL, Tsai MH, Lai SH, Yao TC, Hua MC, Yeh KW, et al. Prenatal exposure to bisphenol-A is associated with Toll-like receptor-induced cytokine suppression in neonates. Pediatric Research. 2016 Mar;79(3):438-44.

149. Liu CH, Xu XJ, Zhang YL, Li WQ, Huo X. Associations between maternal phenolic exposure and cord sex hormones in male newborns. Human Reproduction. 2016 Mar;31(3):648-56.

150. Louis GMB, Barr DB, Kannan K, Chen Z, Kim S, Sundaram R. Paternal exposures to environmental chemicals and time-to-pregnancy: overview of results from the LIFE study. Andrology. 2016 Jul;4(4):639-47.

151. Lv YS, Rui CY, Dai YY, Pang QH, Li YR, Fan RF, et al. Exposure of children to BPA through dust and the association of urinary BPA and triclosan with oxidative stress in Guangzhou, China. Environmental Science-Processes & Impacts. 2016;18(12):1492-9.

152. Maqbool F, Mostafalou S, Bahadar H, Abdollahi M. Review of endocrine disorders associated with environmental toxicants and possible involved mechanisms. Life Sciences. 2016 Jan;145:265-73.

153. Minguez-Alarcon L, Gaskins AJ, Chiu YH, Souter I, Williams PL, Calafat AM, et al. Dietary folate intake and modification of the association of urinary bisphenol A concentrations with in vitro fertilization outcomes among women from a fertility clinic. Reproductive Toxicology. 2016 Oct;65:104-12.

154. Minguez-Alarcon L, Hauser R, Gaskins AJ. Effects of bisphenol A on male and couple reproductive health: a review. Fertility and Sterility. 2016 Sep;106(4): 864-70.

155. Peng FL, Ji WL, Zhu F, Peng DH, Yang M, Liu R, et al. A study on phthalate metabolites, bisphenol A and nonylphenol in the urine of Chinese women with unexplained recurrent spontaneous abortion. Environmental Research. 2016 Oct; 150:622-8.

156. Perez-Lobato R, Mustieles V, Calvente I, Jimenez-Diaz I, Ramos R, Caballero-Casero N, et al. Exposure to bisphenol A and behavior in school-age children. Neurotoxicology. 2016 Mar;53:12-9.

157. Piecha R, Svacina S, Maly M, Vrbik K, Lacinova Z, Haluzik M, et al. URINE LEVELS OF PHTHALATE METABOLITES AND BISPHENOL A IN RELATION TO MAIN METABOLIC SYNDROME COMPONENTS: DYSLIPIDEMIA, HYPERTENSION AND TYPE 2 DIABETES A PILOT STUDY. Central European Journal of Public Health. 2016 Dec;24(4):297-301.

158. Pinson A, Bourguignon JP, Parent AS. Exposure to endocrine disrupting chemicals and neurodevelopmental alterations. Andrology. 2016 Jul;4(4):706-22.

159. Preciados M, Yoo C, Roy D. Estrogenic Endocrine Disrupting Chemicals Influencing NRF1 Regulated Gene Networks in the Development of Complex Human Brain Diseases. International Journal of Molecular Sciences. 2016 Dec; 17(12).

160. Provvisiero DP, Pivonello C, Muscogiuri G, Negri M, de Angelis C, Simeoli C, et al. Influence of Bisphenol A on Type 2 Diabetes Mellitus. International Journal of Environmental Research and Public Health. 2016 Oct;13(10).

161. Tai XC, Chen Y. Urinary bisphenol A concentrations positively associated with glycated hemoglobin and other indicators of diabetes in Canadian men. Environmental Research. 2016 May;147:172-8.

162. Vahedi M, Saeedi A, Poorbaghi SL, Sepehrimanesh M, Fattahi M. Metabolic and endocrine effects of bisphenol A exposure in market seller women with polycystic ovary syndrome. Environmental Science and Pollution Research. 2016 Dec;23(23):23546-50.

163. Vitku J, Heracek J, Sosvorova L, Hampl R, Chlupacova T, Hill M, et al. Associations of bisphenol A and polychlorinated biphenyls with spermatogenesis and steroidogenesis in two biological fluids from men attending an infertility clinic. Environment International. 2016 Apr-May;89-90:166-73.

164. Wang Y, Hollis-Hansen K, Ren X, Qiu Y, Qu W. Do environmental pollutants increase obesity risk in humans? Obesity Reviews. 2016 Dec;17(12): 1179-97.

165. Zhang T, Xue JC, Gao CZ, Qiu RL, Li YX, Li X, et al. Urinary Concentrations of Bisphenols and Their Association with Biomarkers of Oxidative Stress in People Living Near E-Waste Recycling Facilities in China. Environmental Science & Technology. 2016 Apr;50(7):4045-53.

166. Lv YS, Lu SY, Dai YY, Rui CY, Wang YJ, Zhou YX, et al. Higher dermal exposure of cashiers to BPA and its association with DNA oxidative damage. Environment International. 2017 Jan;98:69-74.

167. Liang H, Xu WP, Chen JP, Shi HJ, Zhu J, Liu XQ, et al. The Association between Exposure to Environmental Bisphenol A and Gonadotropic Hormone Levels among Men. Plos One. 2017 Jan;12(1).

168. Milosevic N, Jaksic V, Sudji J, Vukovic B, Icin T, Milic N, et al. Possible influence of the environmental pollutant bisphenol A on the cardiometabolic risk factors. International Journal of Environmental Health Research. 2017 Feb;27(1): 11-26. PubMed PMID: WOS:000393872500002. 169. Wang ZL, Li DK, Miao MH, Liang H, Chen JP, Zhou ZJ, et al. Urine bisphenol A and pubertal development in boys. International Journal of Hygiene and Environmental Health. 2017 Jan;220(1):43-50.
